# N-Type Metal Oxide Semiconductor Hydrogen Sensors: Mechanisms, Materials Design, and Interface Engineering Strategies

**DOI:** 10.3390/nano16120762

**Published:** 2026-06-17

**Authors:** Daewoong Jung

**Affiliations:** 1 Department of Nanomechatronics Engineering, College of Nanoscience & Nanotechnology, Pusan National University, 2 Busandaehak-ro, Busan 46241, Republic of Korea; dwjung@pusan.ac.kr; 2 School of Transdisciplinary Engineering, College of Engineering, Pusan National University, 2 Busandaehak-ro, Busan 46241, Republic of Korea

**Keywords:** hydrogen sensor, n-type metal oxide, ZnO, SnO_2_, In_2_O_3_, chemiresistor, metallization, heterojunction, DFT

## Abstract

Hydrogen is a promising clean-energy carrier, but its low ignition energy, high diffusivity, and wide flammability range demand reliable leak detection. Chemiresistive sensors based on n-type metal oxide semiconductors are attractive owing to their simple architecture, low cost, large resistance modulation, thermal robustness, and compatibility with miniaturized devices. This review focuses on n-type metal oxide semiconductor nanomaterials for hydrogen sensing, particularly ZnO, SnO_2_, In_2_O_3_, WO_3_, TiO_2_, and related mixed oxides. The fundamental sensing mechanisms are examined, including oxygen chemisorption, electron-depletion-layer modulation, grain-boundary barrier control, catalytic hydrogen spillover, and hydrogen-induced surface reduction or metallization, together with the way these mechanisms compete and cooperate under different operating conditions. Recent performance-enhancement strategies are organized around morphology and porosity control, noble-metal sensitization, defect and dopant engineering, n–n heterojunctions, molecular sieving, and low-temperature activation. Density functional theory is discussed as a design tool for evaluating adsorption energetics, vacancy formation, work-function shifts, band alignment, and interfacial charge transfer, along with its current limitations for modeling humid surfaces. Finally, key challenges and future directions, including humidity tolerance, standardized reporting, device integration, and emerging materials, are summarized to guide the development of high-performance hydrogen sensors.

## 1. Introduction

Hydrogen (H_2_) is increasingly used in energy conversion, chemical processing, fuel-cell systems, battery-safety diagnostics, and distributed energy infrastructure. Because its combustion product is water, H_2_ is widely considered a key energy carrier for decarbonization scenarios [1,2,3]. However, the same physicochemical properties that make H_2_ useful also make leakage detection challenging: H_2_ is colorless, odorless, highly diffusive, buoyant, and flammable over a broad concentration range. Therefore, H_2_ sensors must detect leaks well below the lower flammability limit and must operate reliably in air under variable humidity and temperature conditions [4,5].

The U.S. Department of Energy targets frequently cited in the H_2_-sensor literature include detection over approximately 0.1–10% H_2_, operation in ambient air across a broad humidity range, fast response, long service life, miniaturization, and low cost [6,7]. These requirements are difficult to satisfy simultaneously because many sensor materials are strongly influenced by oxygen concentration, humidity, thermal drift, and interfering reducing gases. Optical and electrochemical sensors offer important advantages in specific applications; nevertheless, chemiresistive sensors remain attractive for distributed monitoring because the sensing event can be converted directly into a resistance or conductance change using simple electronics [4,5,6,7,8].

Metal oxide semiconductor (MOS) sensors are a mature subclass of chemiresistive sensors. For H_2_ sensing, n-type oxides such as ZnO, SnO_2_, In_2_O_3_, WO_3_, TiO_2_, and other conductive oxides are particularly important. In n-type MOSs, oxygen adsorption withdraws electrons from the conduction band, forming a surface depletion layer and increasing resistance. When H_2_ reacts with chemisorbed oxygen, electrons are returned to the oxide, narrowing the depletion region and decreasing resistance. Although this basic mechanism is straightforward, practical sensor behavior is governed by coupled surface chemistry, point defects, grain boundaries, morphology, catalytic additives, humidity, and device temperature [9,10,11].

This review deliberately narrows its scope to n-type materials. p-Type oxides are mentioned only when they appear as auxiliary components in composite structures; the primary focus remains on n-type ZnO, SnO_2_, In_2_O_3_, and related n-n heterojunctions. The review also follows an integrated approach to DFT. Instead of presenting DFT as a separate section, computational insights are embedded within the corresponding improvement strategies, reflecting their practical role in materials design: quantifying adsorption energetics, vacancy stability, work-function shifts, and interfacial charge transfer. Device integration is discussed concisely as an application layer rather than as the central materials-design theme.

Several comprehensive reviews of metal oxide semiconductor hydrogen sensors are already available. The present review is distinguished from these works in three respects. First, instead of treating density functional theory (DFT) as a separate methodological section, we embed computational descriptors directly within each materials-design strategy, emphasizing their practical role as a distributed design tool that connects surface energetics to morphology, catalysis, doping, and interface engineering. Second, we deliberately restrict the scope to n-type oxides and treat hydrogen-induced surface metallization as a distinct—though strongly condition-dependent—transduction pathway rather than a universal mechanism. Third, we adopt an application-oriented perspective that extends beyond sensing performance to humidity tolerance, standardized performance reporting, and device integration, which we argue constitute the principal barriers to translating laboratory sensors into reliable hydrogen-safety devices.

### Performance Metrics and Reporting Conventions

A persistent difficulty in comparing H_2_ sensors is the diversity of response definitions, testing atmospheres, and concentration ranges. For n-type reducing-gas sensors, the response is frequently reported as R_a_/R_g_, where R_a_ is the resistance in air and R_g_ is the resistance under H_2_. Other studies use percentage resistance change, conductance ratios, or normalized current change. Response and recovery times are commonly defined as the times required to reach 90% of the total signal change and to return to 10% of the peak signal, respectively [4,6,9]. These conventions must be reported explicitly because response magnitude, apparent detection limit, and recovery kinetics can vary substantially with gas balance, humidity, chamber volume, flow rate, and baseline stabilization protocol. Table 1 summarizes these commonly used performance metrics and highlights the main cautions that should be considered when comparing n-type MOS H_2_ sensors across different studies.

## 2. Fundamentals of N-Type MOS Hydrogen Sensing

### 2.1. Oxygen Chemisorption, Depletion Layers, and Grain-Boundary Barriers

The baseline state of an n-type MOS sensor in air is controlled by oxygen adsorption and ionization. Oxygen molecules adsorb on the oxide surface and extract electrons from the conduction band to form O_2_^−^, O^−^, or O^2−^ species, depending on temperature. This electron withdrawal produces band bending and an electron-depletion layer near the surface. In a polycrystalline film, the depletion layer translates into potential barriers at grain boundaries and necks. Thus, sensor resistance is not simply a bulk material property; it is an emergent function of surface coverage, depletion width, grain size, intergrain-contact geometry, and electrode interfaces [9,10,11].

Upon exposure to hydrogen, H_2_ reacts with chemisorbed oxygen to form water. This reaction releases electrons back to the oxide, reduces band bending, and lowers grain-boundary barriers. In n-type oxides, the resulting conductance increase is measured as the hydrogen response. At low temperatures, insufficient activation of oxygen species or H_2_ dissociation can suppress the response. At excessively high temperatures, desorption and rapid surface turnover can reduce adsorbate coverage, often producing the volcano-shaped temperature dependence reported for MOS sensors [12,13,14]. The representative reaction sequence is as follows [9,10,11,12,13,14,15,16,17,18,19]:

O_2_ (gas) → O_2_ (ads) (1)

O_2_ (ads) + e^−^ → O_2_^−^ (ads) (2)

O_2_ (ads) + 2e^−^ → 2O^−^ (ads) (3)

O^−^ (ads) + e^−^ → O^2−^ (ads) (4)

In this sequence, adsorbed oxygen molecules are converted into ionic oxygen species that extract electrons from the surface of the n-type material. Because electrons are the majority carriers in n-type semiconductors, this charge extraction expands the surface depletion layer, increases the potential barrier for carrier transport, and raises the resistance. When the n-type MOS is exposed to H_2_, the reaction can be described schematically as follows:

H_2_ (gas) → 2H (ads) (5)

H (ads) + O^−^ (ads) → OH^−^ (6)

OH^−^ (ads) + H → H_2_O + e^−^ (7)

**Figure 1:**
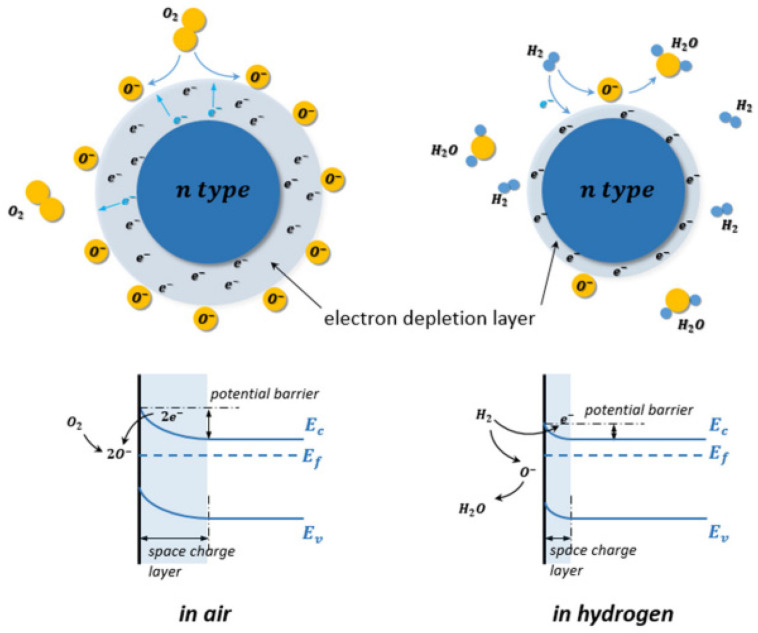
Schematic illustration of the gas sensing mechanism of n-type metal oxide semiconductor and its energy band change in the air and hydrogen. All Panels adapted with permission from Ref. [14].

Figure 1 schematically illustrates the operating principle of an n-type MOS hydrogen sensor. Hydrogen molecules react with oxygen species adsorbed on the n-type MOS surface and are oxidized to water. The electrons previously captured by oxygen species are released back to the conduction band, increasing the surface carrier density, narrowing the depletion layer, and reducing the resistance. In this way, changes in hydrogen concentration can be detected through the corresponding resistance modulation.

**Figure 2:**
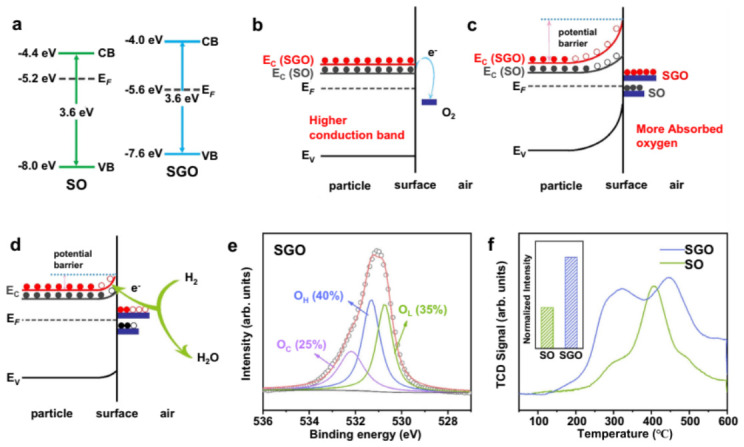
Electronic structure of SGO and SO Sn_0.8_Ge_0.2_O_2_ (named as SGO in the following part, SnO_2_ is named as SO). (**a**) Schematic illustration of the energy band diagram of SO and SGO. Schematic gas sensing mechanism of SO and SGO: in vacuum (**b**), in air (**c**), and exposure to H_2_ (**d**). CB, VB, and EF denote minimum conduction band energy, maximum valence band energy, and Fermi level energy, respectively. The black and red dots represent free electrons in the conduction band of SO and SGO, respectively. (**e**) O 1s XPS spectrum of SGO. (**f**) O_2_-TPD profiles of SO and SGO, with the normalized amount of O_2_ adsorbed onto the surface of SO and SGO (inset), normalized intensity represents O_2_-TPD signal area under 50–600 °C. All Panels adapted with permission from Ref. [11].

These gas reactions can vary substantially depending on the sensing material, its microstructure, and the accessibility of reactive surface sites. Barsan et al. described compact and porous sensing layers as two limiting cases [20]. In compact films, gas interactions occur mainly at the geometric outer surface, whereas in porous films, target gases can penetrate into the sensing layer and react with internal grain surfaces, grain boundaries, necks, and electrode interfaces. In addition to the conventional chemisorbed-oxygen mechanism, recent in situ spectroscopic and DFT studies indicate that surface lattice oxygen can participate directly in hydrogen sensing reactions. The conversion of lattice oxygen into reactive oxygen species, accompanied by surface oxygen-vacancy formation, can further modulate the resistance and enhance the hydrogen response of metal oxide sensors as shown Figure 2 [11].

### 2.2. Hydrogen-Induced Reduction and Metallization

Beyond the generic oxygen-chemisorption mechanism, some n-type oxides can undergo material-specific surface-reduction processes under hydrogen-containing atmospheres. ZnO is the most widely discussed material in this context. First-principles studies have examined hydrogen and oxygen adsorption on ZnO surfaces and nanowires and have predicted that hydrogen adsorption can induce partial metallization of ZnO surfaces, with the extent of this electronic reconstruction depending on surface orientation, curvature, and local coordination environment [21,22,23,24]. Experimentally, electrospun ZnO nanofibers have shown unusually high hydrogen selectivity compared with SnO_2_ nanofibers, and this behavior has often been interpreted in terms of partial surface metallization of ultrafine ZnO nanograins under hydrogen exposure.

The metallization concept is important because it introduces an additional transduction pathway beyond conventional depletion-layer modulation. In the conventional mechanism, hydrogen reacts with chemisorbed oxygen species and returns electrons to the n-type oxide, thereby reducing the surface depletion layer and lowering the resistance. In contrast, hydrogen-induced metallization implies that local reduction or hydrogenation of ZnO can create highly conductive surface states or quasi-metallic conduction pathways. This additional pathway may amplify the resistance change and contribute to the relatively high hydrogen selectivity observed in certain ZnO nanostructures. However, this mechanism should not be regarded as universal for all ZnO-based hydrogen sensors. It is most plausible under specific conditions, such as ultrafine ZnO grains, favorable nonpolar surface terminations, Pd- or Pt-assisted hydrogen dissociation, elevated operating temperatures, and sufficiently reducing local environments.

**Figure 3:**
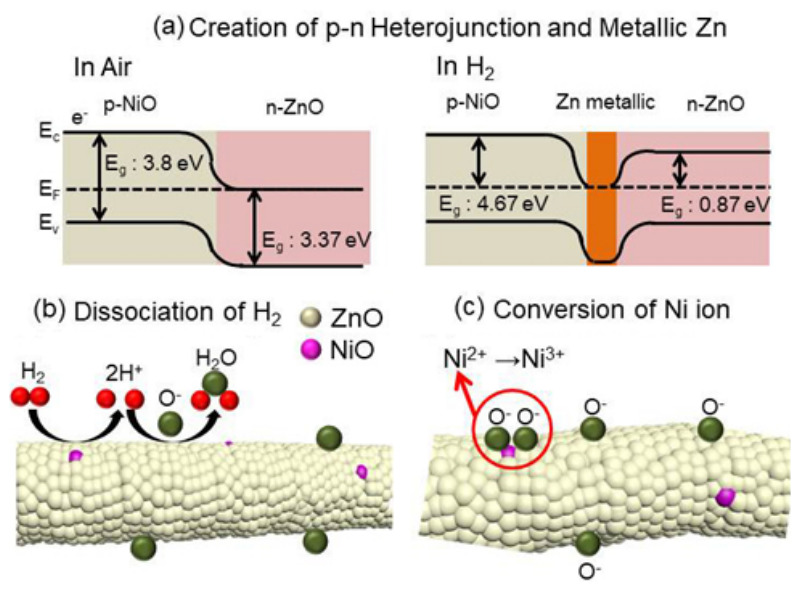
Schematic diagram of sensing mechanism: (**a**) creation of p-n heterojunctions and metallization of ZnO, and (**b**) dissociation and subsequent oxidation of H_2_ gas on the surface of gas sensor, and (**c**) conversion of Ni ions. All Panels adapted with permission from Ref. [25].

Recent experimental studies have provided more direct support for this picture. Lee et al. reported a p-NiO-loaded n-ZnO nanofiber sensor fabricated by electrospinning and proposed that the ZnO surface can be partially reduced to metallic Zn under hydrogen at the sensing temperature [25]. In their interpretation, as illustrated in Figure 3, dissociated hydrogen atoms adsorb on oxygen sites of nonpolar ZnO surfaces, inducing charge delocalization between Zn atoms and O–H bonds. This delocalization partially occupies the Zn 4s and 3d surface states, producing a more conductive surface region in ultrafine ZnO nanograins. When hydrogen is removed and air is reintroduced, the metallic Zn-like surface is reoxidized to ZnO, restoring the original band configuration and baseline resistance. This reversible reduction–oxidation process provides a physically plausible explanation for the large and selective resistance modulation observed in ZnO-based hydrogen sensors.

DFT calculations are particularly useful for evaluating this mechanism when they directly address hydrogen-induced changes in the electronic structure of ZnO. Relevant descriptors include H adsorption energy, H–H dissociation barriers on catalyst-decorated ZnO, oxygen-vacancy formation energy, density-of-states changes near the Fermi level, work-function shifts, and charge-density redistribution at hydrogenated surfaces. Among these, changes in the surface density of states and charge delocalization are especially important for distinguishing true metallization from ordinary electron donation associated with oxygen removal. Nevertheless, computational descriptors should be interpreted together with experimental evidence, because practical sensors operate in complex environments involving oxygen, humidity, grain boundaries, catalyst particles, and metastable hydroxyl species that are difficult to capture fully in idealized slab or nanowire models [26].

It should be emphasized that the evidence for hydrogen-induced metallization of ZnO is largely indirect, being inferred from the unusually high hydrogen selectivity of ultrafine ZnO nanostructures rather than from direct, operando observation of metallic surface states. Comparable resistance modulation can, in principle, also arise from oxygen-vacancy-mediated electron donation, the formation and removal of surface hydroxyl species, or PdH_x_-related effects in catalyst-decorated systems, and these contributions are difficult to deconvolve experimentally. Hydrogen-induced metallization should therefore be regarded as one plausible, condition-dependent contribution—most credible for ultrafine grains, nonpolar surface terminations, Pd/Pt-assisted dissociation, and sufficiently reducing local environments—rather than a general feature of all ZnO-based hydrogen sensors.

### 2.3. Debye Length, Grain Size, and Utility Factor

**Figure 4:**
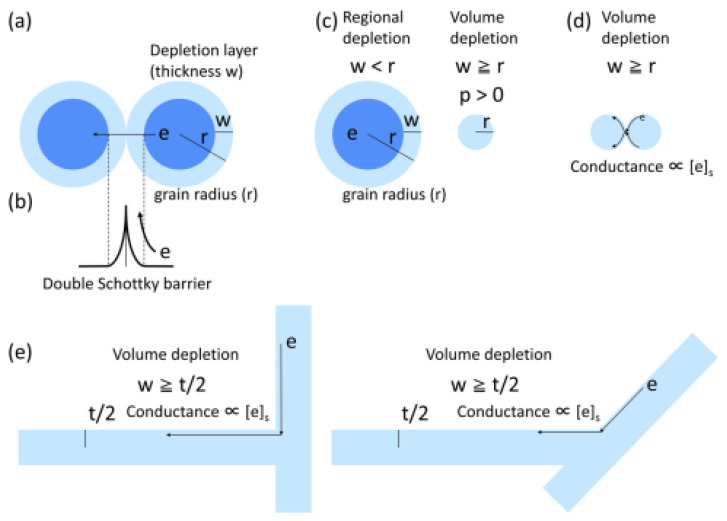
Diagrams of electron depletion for oxide grains and the resistance of contact between grains. (**a**) Space charge layer model, (**b**) double Schottky barrier model, (**c**) regional and volume depletion model, (**d**) surface conductive grains contact model, (**e**) volume depletion model for the dendric growth of thin nanosheet. All Panels adapted with permission from Ref. [30].

The magnitude of the chemiresistive response is maximized when a substantial portion of the electrical conduction pathway is affected by surface charge modulation. This condition is commonly described in terms of the relationship between the grain size and the Debye length. Figure 4 schematically illustrates electron depletion in oxide grains and the contact resistance formed at grain boundaries. When the grain radius is much larger than the surface depletion width, only a thin shell near the surface is modulated by gas adsorption and reaction, while the conductive core remains largely unaffected. As a result, the resistance change is limited. In contrast, when the grain size becomes comparable to, or smaller than, the Debye length, the entire grain can be depleted in air and strongly modulated upon exposure to hydrogen. This size-dependent modulation explains why nanocrystalline grains, hollow architectures, and electrospun nanofibers often show higher responses than dense thick films [11,20,26,27,28,29,30].

The utility factor introduces an additional design constraint by describing how effectively the sensing layer participates in the gas reaction and signal transduction. A thick sensing layer may contain a large number of active sites; however, if hydrogen is consumed, delayed, or blocked near the outer surface, the inner region contributes little to the overall response. Porous structures can improve gas penetration and increase the accessible surface area, but excessive porosity may interrupt percolated conduction pathways and weaken electrical connectivity. Therefore, the optimal sensing body is not necessarily the material with the highest BET surface area. Rather, it is the structure that achieves an appropriate balance among accessible active sites, continuous electron-transport pathways, efficient gas diffusion, and thermal stability [29]. In this respect, MOF-derived oxides, electrospun nanofibers, and sputtered porous films are attractive platforms because their morphology, porosity, grain connectivity, and composition can be adjusted with relative flexibility [27,28].

### 2.4. Interplay of Sensing Mechanisms

In a working sensor, the mechanisms described above rarely operate in isolation. Oxygen chemisorption [9,10,11], catalytic spillover [12,18,19] heterojunction barrier modulation [23,24,31], hydrogen-induced metallization [25,32,33], and defect-mediated transduction [34,35,36] coexist within the same sensing layer, and their relative contributions shift with operating temperature, humidity, hydrogen concentration, catalyst loading, and microstructure. Identifying which mechanism dominates under a given set of conditions is therefore essential both for interpreting reported performance and for rational design. This subsection summarizes the relative contributions of the principal mechanisms and the conditions under which each becomes dominant; Table 2 collects, for each mechanism, the dominant operating regime, the primary contribution to the signal, an experimentally or computationally accessible diagnostic signature, and representative material systems.

Oxygen chemisorption provides the universal baseline transduction common to all n-type oxides and is most effective at intermediate operating temperatures, where ionosorbed oxygen species are activated but not yet desorbed, giving rise to the characteristic volcano-shaped response–temperature relationship. At lower temperatures, catalytic spillover from noble-metal nanoparticles becomes the principal route to a measurable response, because it lowers the effective activation energy for hydrogen dissociation and surface reaction; its contribution scales with catalyst dispersion and the metal/oxide perimeter rather than with bulk loading. In composite and bilayer architectures, heterojunction barrier modulation adds an interfacial contribution that amplifies the resistance change beyond that of single-oxide layers, and its magnitude increases with interface density and therefore with decreasing grain size. Hydrogen-induced metallization is the most condition-restricted contribution: it is credible only for ultrafine ZnO grains with favorable surface terminations under sufficiently reducing, catalyst-assisted conditions, and even then it is difficult to separate experimentally from oxygen-vacancy donation, surface-hydroxyl chemistry, or PdH_x_-related effects (Section 2.2). Defect engineering acts as a cross-cutting modifier: oxygen vacancies and aliovalent dopants tune the baseline conductivity, the density of adsorption-active sites, and the width of the depletion layer, and their benefit is generally confined to an optimum concentration window beyond which excess carriers suppress the relative response.

These mechanisms can act synergistically or in competition. In Pd-functionalized In_2_O_3_-ZnO [31] or Pd@ZnO/SnO_2_ [32] systems, for example, spillover, heterojunction modulation, and—under suitable conditions—ZnO metallization can reinforce one another, whereas excessive catalyst loading or over-doping can raise the baseline conductivity and thereby diminish the relative contribution of surface depletion. The net response is further constrained by the utility factor and by percolation in porous layers (Section 2.3): a mechanism that strengthens the local surface signal contributes little to the measured resistance if the affected region is poorly connected to the conduction path or is inaccessible to the analyte. Consequently, the relative weighting summarized in Table 2 should be read as a guide to the dominant contribution under typical conditions rather than as a fixed hierarchy, and disentangling coexisting mechanisms in a specific device generally requires complementary characterization such as temperature-dependent response analysis, impedance spectroscopy, work-function measurements, and operando spectroscopy.

## 3. N-Type Materials for Hydrogen Sensors

**Figure 5:**
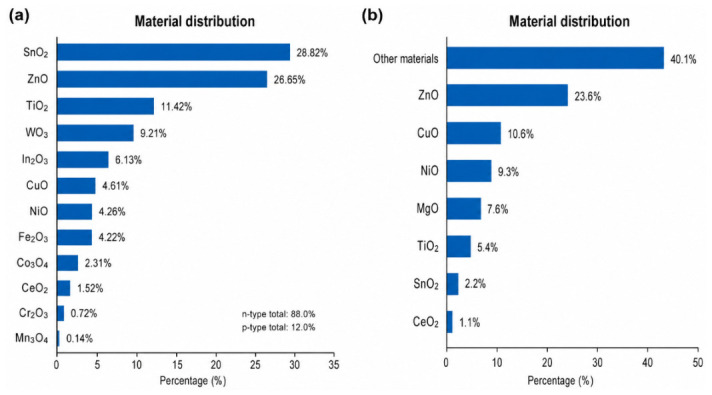
(**a**) Studies on oxide semiconductor gas sensor and (**b**) the proportion of different metal oxides published as sensitive materials for hydrogen sensors.

This section defines the material scope of this review. Figure 5 summarizes the publication trends of oxide semiconductor gas sensors and the relative proportions of metal oxides reported as hydrogen-sensing materials. N-type metal oxides have been more extensively investigated for chemiresistive gas sensing than p-type oxides, mainly because of their relatively high electron mobility, stable baseline conductivity, rich surface oxygen chemistry, and compatibility with diverse nanostructuring and device-integration processes. Among them, SnO_2_ and ZnO have received particularly strong attention as representative gas-sensing materials [30]. In the specific context of hydrogen sensing, the literature is especially rich for ZnO and SnO_2_, followed by TiO_2_, In_2_O_3_, WO_3_, and other n-type oxide systems [15]. The advantages, limitations, and common improvement routes of representative n-type metal oxide families for hydrogen sensing are summarized in Table 3. Accordingly, this review focuses primarily on SnO_2_- and ZnO-based hydrogen sensors, while In_2_O_3_, TiO_2_, and WO_3_ are discussed more concisely as important sensing materials, catalytic supports, and heterojunction components. This scope allows the review to emphasize both the dominant material platforms and the broader interface-engineering strategies that govern hydrogen response, selectivity, and operating temperature.

### 3.1. SnO_2_-Based Hydrogen Sensors

**Figure 6:**
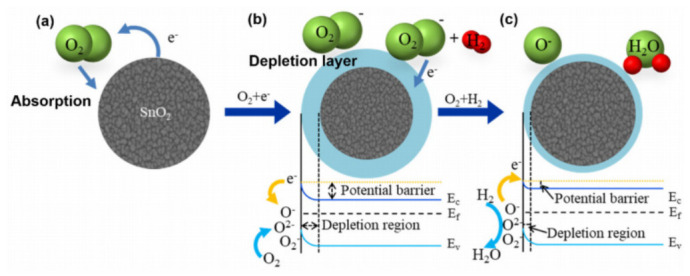
N-type metal oxide hydrogen sensing mechanism; (**a**) SnO_2_ as-deposited; (**b**) SnO_2_ in air; (**c**) SnO_2_ in H_2_ reduction gas. All Panels adapted with permission from Ref. [44].

SnO_2_ is one of the prototypical n-type metal oxides used in commercial chemiresistive gas sensors. It possesses a rutile crystal structure, a wide band gap, good thermal and chemical stability, and abundant surface oxygen-related defect sites, all of which make it suitable for resistance-based gas transduction. SnO_2_-based hydrogen sensors have therefore been investigated in various forms, including nanoparticles, nanowires, nanotubes, nanofibers, hollow spheres, porous films, sputtered thin films, and multicomponent composites [17,18,42,43,44,45]. Figure 6 schematically illustrates the hydrogen-sensing mechanism of SnO_2_ under as-deposited, air-exposed, and H_2_-reduction conditions.

**Figure 7:**
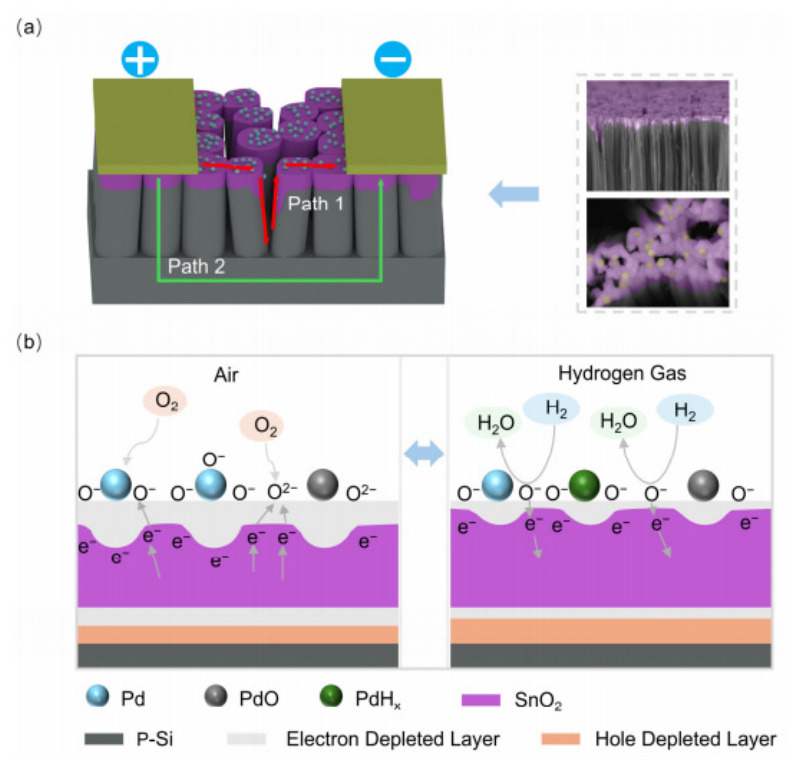
(**a**) The overall structure and electrical circuit; (**b**) gas sensing mechanism. All Panels adapted with permission from Ref. [47].

Despite these advantages, pristine SnO_2_ generally requires elevated operating temperatures to achieve a high hydrogen response, typically in the range of 200–400 °C. In addition, because its sensing mechanism relies strongly on surface oxygen chemisorption, SnO_2_ can respond to a broad range of reducing gases, and its baseline resistance and sensitivity are often strongly affected by humidity. Consequently, much of the SnO_2_ hydrogen-sensor literature has focused on surface functionalization, catalyst loading, morphology control, and composite formation as routes to improve response, selectivity, and operating temperature [18,46]. Figure 7 schematically shows the overall device structure, electrical circuit, and gas-sensing mechanism of the Pd-decorated SnO_2_ nanofilm sensor [47]. For example, Au-loaded SnO_2_ nanoparticles have been reported for low-concentration hydrogen detection [48], whereas Pd- or Pt-loaded SnO_2_ structures exploit noble-metal-catalyzed hydrogen dissociation, spillover, and electronic sensitization at metal/oxide interfaces [49]. More recently, MOF-derived porous Pd@SnO_2_ composites have been explored for room-temperature hydrogen sensing, highlighting the importance of combining accessible porous architectures with highly dispersed catalytic sites [12].

Multicomponent n-type composites provide an additional route to enhance SnO_2_-based hydrogen sensing by coupling surface reaction kinetics with interfacial charge modulation. A Pd/ZnO/SnO_2_ hollow-nanofiber sensor incorporating ternary Pd/ZnO/SnO_2_ heterojunctions, fabricated by electrospinning and magnetron sputtering, has been reported to exhibit high response, a low detection limit, good selectivity, and improved repeatability [49]. Similarly, SnO_2_–In_2_O_3_, SnO_2_–ZnO, and SnO_2_–TiO_2_ systems can tune band alignment, oxygen-vacancy concentration, carrier transport, and surface reaction kinetics [23,24,50,51]. These examples indicate that the role of SnO_2_ in hydrogen sensors is not limited to serving as a single-component sensing layer; it can also act as a stable transduction backbone in catalyst-decorated and heterojunction-based sensor architectures.

### 3.2. ZnO-Based Hydrogen Sensors

**Figure 8:**
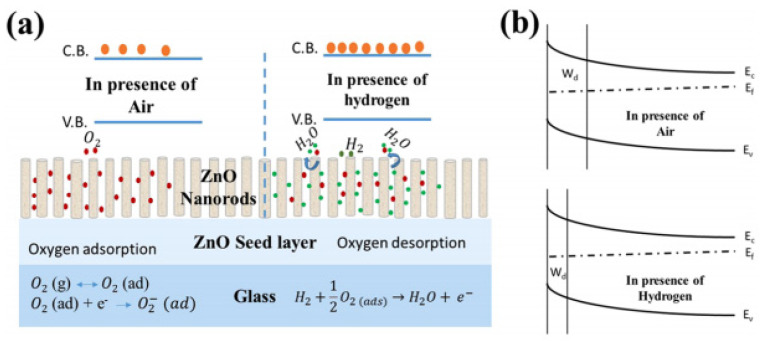
(**a**) Schematic diagram explaining the H_2_-sensing mechanism of sensors based on a metal oxide semiconductor in the presence of air and hydrogen gas together with the respective adsorption processes of oxygen and its reaction with hydrogen, generating conduction band electrons and (**b**) Energy band diagrams in presence of air and hydrogen, explaining the reduction in depletion width Wd. All Panels adapted with permission from Ref. [55].

ZnO is a wide-band-gap n-type semiconductor with a stable wurtzite structure, high electron mobility, low toxicity, and excellent morphological tunability. It can be synthesized in diverse forms, including nanowires, nanorods, nanotubes, nanofibers, nanosheets, nanoflowers, and thin films, using hydrothermal growth, sol–gel processing, electrospinning, sputtering, vapor transport, and related methods [52]. Since the early report by Seiyama et al. that the resistance of ZnO films changes upon gas adsorption and desorption [52], ZnO has been widely investigated as a representative gas-sensing material. In hydrogen sensing, ZnO is particularly attractive because surface defects, polar facets, high surface-to-volume ratios, and hydrogen-induced surface metallization can produce strong conductance modulation [32,53,54,55]. Figure 8 schematically illustrates the oxygen adsorption process in air, the reaction with hydrogen, and the resulting reduction in depletion width in a ZnO based-gas sensor [55].

Despite these advantages, pristine ZnO sensors rarely satisfy practical requirements for selectivity, operating temperature, and humidity tolerance. ZnO nanowires and nanorods often respond not only to H_2_ but also to other reducing gases, such as ethanol, acetone, CO, and methane, and their baseline and response can be strongly affected by humidity [56]. Size effects are also significant. Individual ZnO nanowires with smaller diameters have been reported to exhibit stronger room-temperature hydrogen response and selectivity than larger-diameter nanowires, which is consistent with enhanced surface-to-volume ratio, more effective depletion-layer modulation, and defect-mediated transduction. Electrospun ZnO nanofibers are a notable case among ZnO nanostructures because their ultrafine nanograins can promote hydrogen-induced surface metallization, leading to unusually high hydrogen selectivity compared with SnO_2_ nanofibers [33]. However, this metallization effect should be considered a condition-dependent enhancement mechanism rather than a universal feature of all ZnO sensors. The limitations of this interpretation, and the alternative explanations that can reproduce similar behavior, are discussed in Section 2.2.

**Figure 9:**
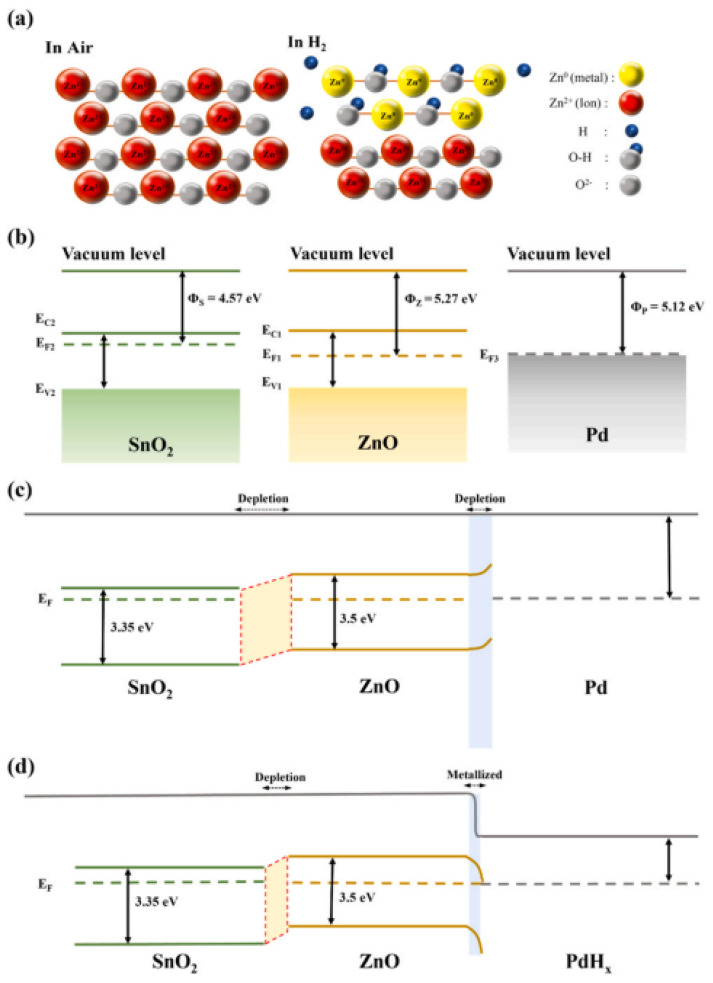
Sensing mechanism of Pd@ZnO/SnO_2_ heterojunction hydrogen sensor (**a**) ZnO metallization mechanism Energy bands of SnO_2_, ZnO, Pd (**b**) before contact to the air, after contact (**c**) in air, (**d**) in hydrogen. All Panels adapted with permission from Ref. [32].

Recent studies have extended this metallization concept from nanofiber networks to heterojunction thin-film platforms. For example, the Pd@ZnO/SnO_2_ bilayer sensor reported by Ha et al. combines several functions in one architecture: Pd promotes hydrogen dissociation and spillover, ZnO provides a metallization-sensitive surface, and SnO_2_ contributes stable charge transport and heterointerface modulation [32]. This type of design illustrates how ZnO-specific surface chemistry can be integrated with catalytic and interfacial engineering to improve hydrogen response over a wide concentration range. Figure 9 schematically illustrates the Pd@ZnO/SnO_2_ heterojunction sensing mechanism, including Pd-assisted hydrogen dissociation, SnO_2_/ZnO interfacial modulation, and hydrogen-induced ZnO metallization.

Noble-metal decoration remains the most widely used strategy for improving ZnO-based hydrogen sensors. Pd, Pt, Au, Ag, and bimetallic nanoparticles provide catalytic sites for H_2_ dissociation and can also form Schottky contacts with ZnO, thereby modifying the surface depletion layer and interfacial barrier height [37,57,58,59]. Pd is particularly effective because it promotes hydrogen dissociation and can introduce PdH_x_-related selectivity. Accordingly, Pd-functionalized ZnO nanowires have shown much higher hydrogen selectivity than bare ZnO nanowires [37]. Bimetallic catalysts provide an additional degree of freedom. For instance, Ag–Pd nanoparticles decorated on ZnO nanorods have been reported to improve hydrogen selectivity over CO, CO_2_, and NH_3_ more effectively than monometallic Au systems, which can be attributed to modified adsorption energetics and catalytic activity at bimetallic surfaces [60]. Similarly, PdPt@ZnO core–shell nanoparticles demonstrated selective hydrogen detection by combining morphology control with the catalytic activity of alloyed PdPt cores [61].

**Figure 10:**
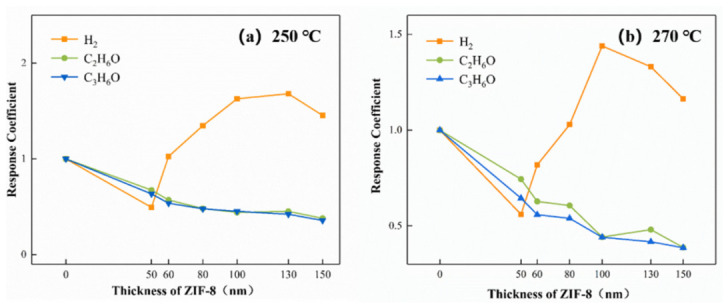
The response coefficients vs. the thicknesses of the ZIF-8 curve at (**a**) 250 °C and (**b**) 270 °C. All Panels adapted with permission from Ref. [64].

ZnO-based composites further expand the design space by introducing homojunctions, n–n heterojunctions, and multiple catalytic interfaces. In composite oxides, smaller grains can increase the number of junctions and active interfaces, thereby enhancing barrier modulation and enabling ppb-level hydrogen detection at elevated temperatures [32,62,63]. In_2_O_3_-loaded ZnO nanofibers and Pd-functionalized In_2_O_3_–ZnO nanofibers show strong enhancement because ZnO, In_2_O_3_, and Pd provide complementary functions: ZnO contributes hydrogen-sensitive surface modulation, In_2_O_3_ modifies carrier transport and interfacial band alignment, and Pd accelerates hydrogen dissociation [31]. In addition to electronic and catalytic modification, physical filtering can improve selectivity. Molecular-sieving ZnO@ZIF-8 architectures allow small H_2_ molecules to diffuse through the porous shell while suppressing the access of larger volatile organic compounds, thereby improving hydrogen selectivity [64]. Figure 10 shows how the ZIF-8 shell thickness influences the response coefficient of ZnO@ZIF-8 core–shell sensors at different operating temperatures.

Overall, ZnO is not merely a conventional oxygen-chemisorption-type sensing oxide. Its hydrogen response can be amplified by a combination of nanoscale depletion-layer modulation, defect-mediated adsorption, noble-metal-catalyzed spillover, heterojunction barrier control, and, under suitable conditions, hydrogen-induced surface metallization. This combination of mechanisms explains why ZnO remains one of the most important platforms for n-type MOS hydrogen sensors, while also emphasizing the need for careful control of catalyst loading, grain size, humidity effects, and operating temperature.

### 3.3. Other N-Type MOS-Based Hydrogen Sensors (In_2_O_3_, WO_3_, and TiO_2_)

In_2_O_3_ is an n-type oxide with relatively high electron mobility and strong sensitivity to surface oxygen defects, making it an attractive platform for chemiresistive hydrogen sensing. In_2_O_3_ nanosensors have often been interpreted using Schottky-junction and thermionic-emission models, reflecting the importance of interfacial barriers and temperature-dependent carrier transport in their sensing behavior. Pd-loaded In_2_O_3_ microspheres and La/Pd co-sensitized In_2_O_3_ nanotubes further illustrate how catalytic activation and defect engineering can lower the operating temperature and improve the hydrogen response [65,66,67,68].

Recent studies on In_2_O_3_-based sensors have increasingly focused on dopant-controlled oxygen vacancies, cation-orbital hybridization, and band-structure modulation [69,70,71]. Rare-earth-doped In_2_O_3_ nanofibers, Ag-modified Tb-doped double-phase In_2_O_3_, and CeO_2_-loaded In_2_O_3_ hollow spheres have been used to tune the electronic structure, surface defect chemistry, and reaction energetics of hydrogen and oxygen species [69,70].

**Figure 11:**
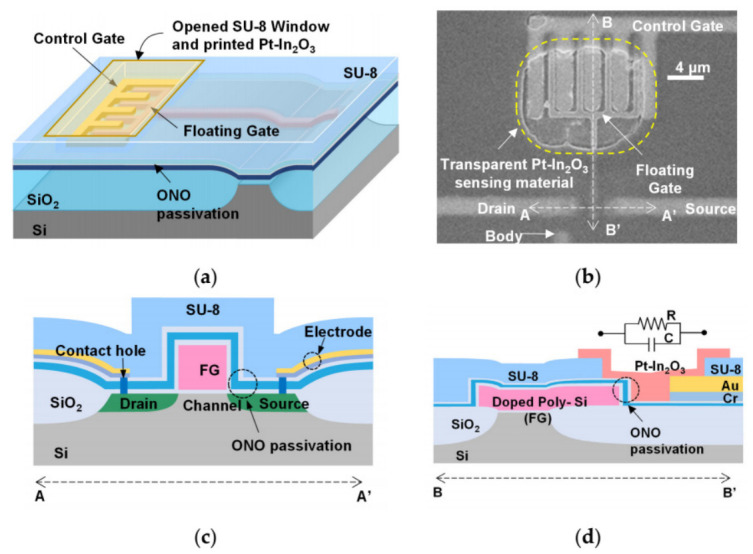
SEM image and schematic views of FET-type sensors. (**a**) Schematic diagram of the 3D structure of the sensor. (**b**) SEM top view of the sensor. The transparent Pt-In_2_O_3_ sensing material is deposited on top of the coupling region of the CG and the FG, which is enclosed by the yellow frame. (**c**,**d**) Schematic cross-sectional views of the sensor along A-A′ and B-B′ in (**b**), respectively. Figure 1. SEM image and schematic views of FET-type sensors. All Panels adapted with permission from Ref. [71].

For example, a room-temperature FET-type H_2_ sensor based on Pt-decorated In_2_O_3_ nanoparticles has been demonstrated using an inkjet-printed sensing gate on a floating-gate FET platform. Figure 11 shows the SEM image and schematic structure of the Pt–In_2_O_3_ FET-type H_2_ sensor, highlighting the integration of the sensing material on the floating-gate device platform. The sensing enhancement was attributed to Pt-assisted H_2_ dissociation, H_2_-induced work-function modulation of the Pt–In_2_O_3_ gate, and the intrinsic signal amplification of the FET device structure [71].

Zn-doped In_2_O_3_ porous nanospheres synthesized by a solvothermal method exhibited enhanced H_2_ sensitivity. The introduction of non-precious-metal Zn as a dopant increased the surface activity of In_2_O_3_, producing a response of 24.6 toward 500 ppm H_2_ at 340 °C, which was substantially higher than that of pure In_2_O_3_. The 2% Zn–In_2_O_3_ sensor also showed ultrafast response/recovery behavior of 2/3 s and a low detection limit of 100 ppb [34]. Bimetallic In_2_O_3_-based materials are also emerging as promising n-type sensing platforms because their electronic levels, defect density, and surface adsorption properties can be regulated by the cation ratio and porous architecture. For instance, an Ag/Cu–In_2_O_3_ sensor exhibited a response of 9.66 toward 100 ppm H_2_ at an optimal operating temperature of 300 °C, together with good reproducibility, selectivity, and long-term stability [35]. Such mixed-cation oxide systems are particularly attractive for DFT-guided screening because cation composition directly affects band-edge positions, oxygen-vacancy stability, H/O adsorption energetics, and interfacial charge transfer.

**Figure 12:**
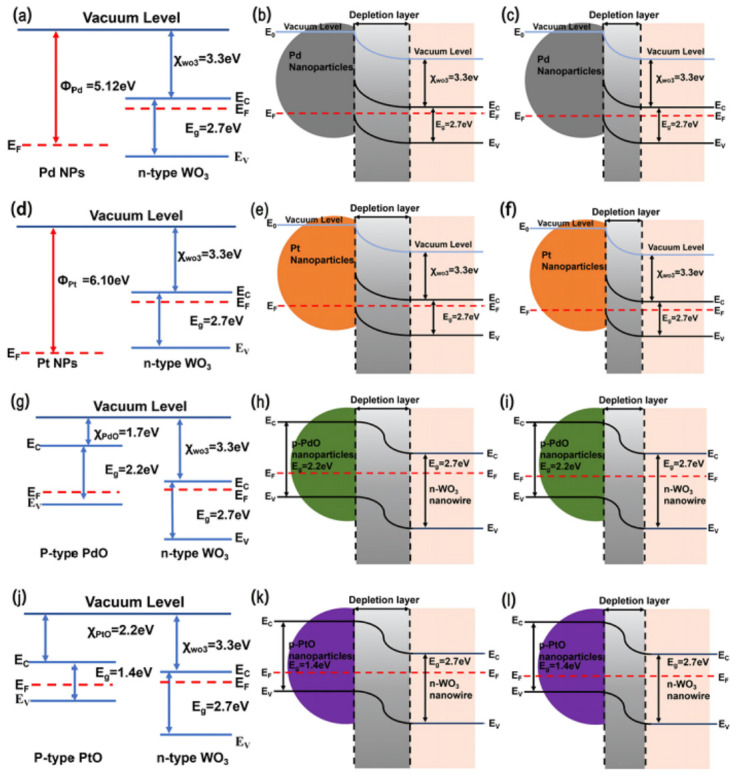
Schematic energy-band diagrams illustrating the interfacial electronic modulation mechanism of the Pt–Pd–WO_3_ nanofiber sensor. (**a**–**c**) Energy-band diagrams of the Pd/WO_3_ interface before contact, after contact in air, and after exposure to H_2_, respectively. (**d**–**l**) The same scenario for the Pt/WO_3_ interface, p-PdO/n-WO_3_ heterojunction and the p-PtO/n-WO_3_ heterojunction, respectively. All Panels adapted with permission from Ref. [73].

WO_3_ and TiO_2_ are also important in hydrogen-sensor design, even when they are not used as the primary sensing materials. WO_3_ provides redox-active surfaces and can participate in n–n heterojunctions with In_2_O_3_ or SnO_2_, thereby modifying carrier transport and surface reaction kinetics [39,72]. In recent work, Pt/PtO and Pd/PdO_x_ co-decorated WO_3_ nanofibers were prepared via electrospinning and calcination for ppb-level H_2_ detection. The optimized 2 at% Pt–2 at% Pd-decorated WO_3_ nanofiber sensor reliably detected 100 ppb H_2_ at 170 °C and showed good linearity, selectivity, repeatability, and long-term stability. The interfacial electronic modulation mechanism of the Pt/PtO- and Pd/PdO_x_-decorated WO_3_ nanofiber sensor is schematically illustrated in Figure 12. The enhanced H_2_-sensing performance was attributed to the porous one-dimensional WO_3_ nanofiber network, catalytic H_2_/O_2_ activation and possible spillover effects of Pt/PtO and Pd/PdO_x_, PdH_x_-related modulation, and interfacial depletion-layer modulation at Pt/PtO–WO_3_ and Pd/PdO_x_–WO_3_ heterostructures [73].

**Figure 13:**
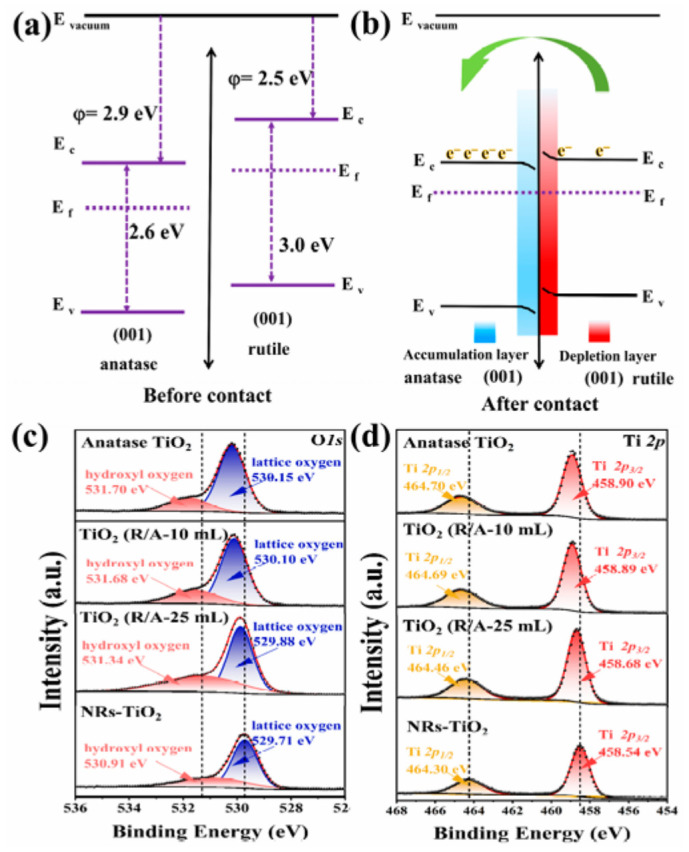
(**a**,**b**) Bandgaps (Eg) and the energies of the valence band (Ev) and conduction band (Ec) for the rutile and anatase phases of titania. (**c**) O1s and (**d**) Ti2p XPS profiles of the NR-TiO_2_ and TiO_2_ (R/A-25 mL) samples. All Panels adapted with permission from Ref. [76].

TiO_2_ is chemically stable and can form effective Schottky contacts with noble metals such as Pd, Au, and Pt [74,75]. Although its intrinsic conductivity is lower than that of many other n-type oxides, TiO_2_ can serve as a robust support or interface-modulating component in hydrogen sensors. For example, a dual-phase rutile–anatase TiO_2_ homojunction material has been prepared for hydrogen sensing. The band alignment and XPS characteristics of the rutile–anatase TiO_2_ homojunction are summarized in Figure 13. The interface between the rutile and anatase nanocrystalline phases formed a uniform homojunction, while the porous structure facilitated carrier transport and gas diffusion. These combined effects enhanced the hydrogen response of the sensor [76].

### 3.4. Material Comparison

Beyond intrinsic sensing performance, the choice of oxide platform depends strongly on the target application and on factors such as process compatibility and thermal and long-term stability. Table 4 summarizes the suitability of representative platforms for four common application paradigms—low-power MEMS micro-hotplate sensors, disposable or printed sensors, high-temperature industrial monitors, and room-temperature safety alarms. Broadly, sputtered SnO_2_ and SnO_2_-based heterojunction thin films are well matched to CMOS/MEMS integration and high-temperature stability; ZnO nanofibers and nanorods offer high sensitivity and selectivity but are more challenging to integrate as patterned thin films; and In_2_O_3_ provides low-temperature, high-mobility operation but requires management of baseline drift. The integration aspects underlying this comparison are discussed further in Section 5.

**Table 3:** Comparison of representative n-type metal oxide families for hydrogen sensing.

Material	Advantages for H_2_ Sensing	Typical Limitations	Common Improvement Route	Representative References
SnO_2_	Mature commercial base, strong oxygen chemisorption, high stability, sputtering/MEMS compatibility	High operating temperature, response to many reducing gases, humidity effects	Pd/Au/Pt sensitization, porous or hollow structures, ZnO/In_2_O_3_ composites, hydrophobic coatings	[12,15,16,17,18,19,24,42,43,44,45,46,47,48,49,50,51]
ZnO	High electron mobility, rich nanostructures, H-induced metallization, facile noble-metal functionalization	Poor pristine selectivity, humidity dependence, high temperature for many structures	Pd/Pt/Au decoration, Ni/Co/Cd doping, SnO_2_ or In_2_O_3_ composites, ZIF-8 sieving	[31,32,33,37,52,53,54,55,56,57,58,59,60,61,62,63,64]
In_2_O_3_	High mobility, defect-sensitive surface, transparent conductive integration potential	High carrier density may reduce relative response; humidity and drift require control	Pd loading, La/Tb/Zn/Ga doping, porous MOF-derived structures, ZnO composites	[34,35,65,66,67,68,69,70,71]
WO_3_	Redox-active surface, useful n-n junction component	Often requires thermal activation; selectivity must be engineered	WO_3_/In_2_O_3_ or WO_3_/PdO junctions, noble-metal sensitization, oxygen-vacancy control	[39,72,73]
TiO_2_	Chemical stability, strong support for Pd catalysts, nanotube architectures	Lower intrinsic conductivity; response can be slow without catalysts	Metal/TiO_2_ Schottky junctions, nanotubes, external activation	[74,75,76]

**Table 4:** Suitability of representative n-type oxide platforms for different hydrogen-sensor application paradigms.

Application Paradigm	Recommended Materials and Morphology	Catalyst/Interface Strategy	Process Compatibility and Integration	Thermal and Long-Term Stability [Ref.]
Low-power MEMS micro-hotplate	Sputtered/ALD SnO_2_ and SnO_2_-based heterojunction thin films; thin, moderately porous films	Pd/Pt sensitization; SnO_2_/ZnO bilayer	Directly CMOS/MEMS-compatible; reproducible; localized deposition on suspended membranes feasible	Good high-temperature stability; manage catalyst sintering [32,39]
Disposable/printed sensors	Solution-processable ZnO and In_2_O_3_ nanostructures, MOF-derived oxides; printable inks	Pd/Au decoration; molecular-sieve coatings	Inkjet/screen/aerosol-jet printing on flexible substrates; low thermal budget	Moderate stability, sufficient for single or short-term use [12,41,52]
High-temperature industrial monitors	SnO_2_, WO_3_, and robust n–n(p) heterojunctions; dense or hierarchical structures	Oxygen-vacancy and heterojunction engineering; stable supports	Compatible with refractory electrodes/packaging; sputtering preferred	High intrinsic thermal stability; resistance to sintering and drift required [39,72,73]
Room-temperature safety alarms	ZnO/In_2_O_3_ nanofibers and nanorods, Pd@SnO_2_ porous composites, TiO_2_ homojunctions; high-surface-area morphologies	Single-atom/Pd catalysts; photoactivation; molecular sieving	Often electrospinning/hydrothermal → integration challenge (see Section 5)	Manage humidity drift and slow recovery at room temperature [12,31,49,66,76]

## 4. Materials and Interface Engineering Strategies

The following sections organize the major performance-improvement strategies for n-type MOS hydrogen sensors, including morphology and porosity control, noble-metal sensitization, defect and dopant engineering, n–n heterojunction formation, and low-temperature activation. DFT and related first-principles calculations are discussed within these strategies as design tools rather than treated as an independent topic. This organization emphasizes that computational descriptors are most useful when they are directly connected to experimentally relevant design factors, such as surface reaction energetics, oxygen-vacancy formation, hydrogen dissociation, interfacial charge transfer, band alignment, and work-function modulation.

### 4.1. Morphology, Porosity, and Mass Transport

Morphology engineering is one of the most effective strategies for improving n-type MOS hydrogen sensors because it increases the number of accessible receptor sites and shortens gas-diffusion pathways [18,19,25,28,29,42]. One-dimensional nanowires and nanorods provide direct electron-transport pathways together with high surface-to-volume ratios, whereas electrospun nanofibers form porous, percolated networks composed of interconnected oxide grains. Hollow spheres and MOF-derived oxides further increase the accessible surface area by providing internal cavities, mesopores, and open diffusion channels. These structural features enhance the probability that hydrogen molecules reach reactive surface sites and that the resulting surface charge modulation is efficiently transferred into a measurable resistance change. 

**Figure 14:**
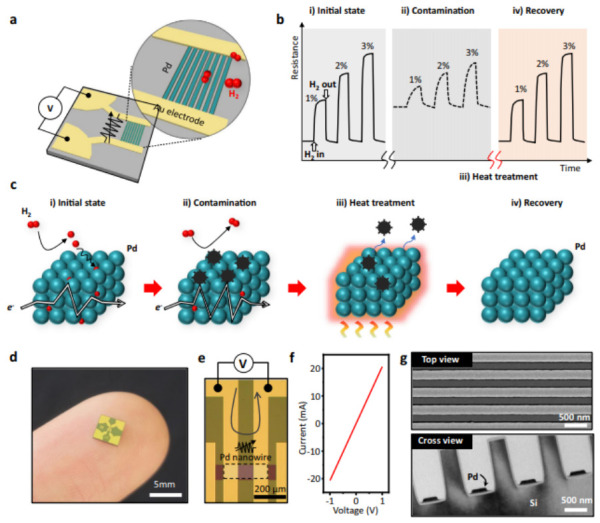
(**a**) Schematic illustration of the Pd nanowire H_2_ sensor. (**b**) Concept of hydrogen sensor performance recovery by applying heat treatment. (**c**) Detailed schematic diagram of degraded performance due to the contaminations (black) of Pd hydrogen sensor and recovery by heat treatment. (**d**) Optical image of Pd nanowire H_2_ sensor. (**e**) Schematic diagram of the 2-point electrical measurements with Au electrodes (yellow) and Pd nanowires (red) (**f**) I–V curve of the Pd nanowire H_2_ sensor. (**g**) Scanning electron microscope (SEM) images for top view and cross view. All Panels adapted with permission from Ref. [77].

The importance of dimensional control is particularly evident in diameter-dependent nanowire sensors. Reducing the characteristic dimension can increase the response and selectivity by allowing a larger fraction of the conduction channel to be affected by surface reactions and depletion-layer modulation [33,38,69]. Kim et al. [77] showed that reducing Pd into nanowire geometries enhances H_2_ sensing because nanoscale dimensions increase the surface-to-volume ratio, promote hydrogen adsorption and diffusion into Pd, and strengthen electron scattering induced by absorbed hydrogen atoms. Figure 14 shows the structure, electrical configuration, and sensing-performance recovery concept of the Pd nanowire-based H_2_ sensor.

In their Pd nanowire sensor, the resistance change was directly related to hydrogen-induced scattering within the Pd conduction pathway, while the nanowire geometry enabled rapid and sensitive room-temperature detection.

**Figure 15:**
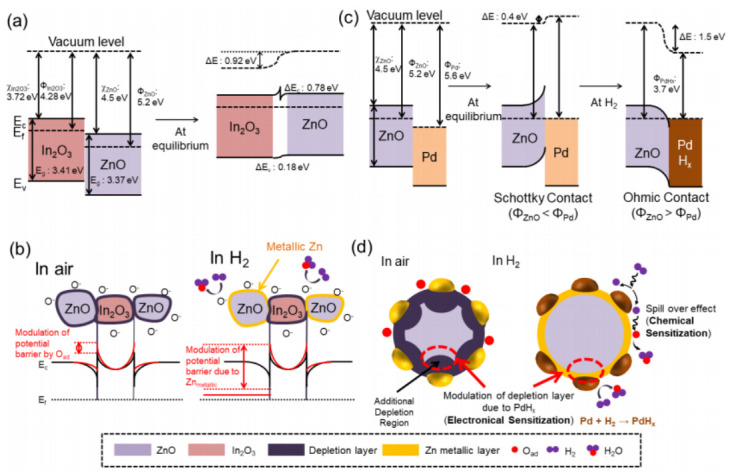
(**a**) Energy levels of In_2_O_3_ and ZnO before and after contact in a vacuum. (**b**) Energy levels of ZnO and In_2_O_3_ after contact in air and hydrogen atmospheres. (**c**) Energy levels of Pd and ZnO before and after contact in air and hydrogen atmospheres. (**d**) Electronic and chemical sensitization effects of Pd. All Panels adapted with permission from Ref. [31].

Composite nanomaterials provide an additional advantage because mesoporosity can accelerate gas diffusion, while heterointerfaces can modify band alignment, carrier transport, and surface reaction kinetics [23,31,54,58,78]. For example, the energy-level alignment and sensitization effects in the Pd-functionalized In_2_O_3_/ZnO system are schematically shown in Figure 15 [31]. Therefore, morphology control should be considered together with interface engineering rather than treated only as a surface-area enhancement strategy.

A key design trade-off is the balance between accessible surface area and electrical connectivity. Highly porous materials can exhibit large responses because of their abundant active sites and open diffusion pathways, but excessive porosity may disrupt percolated conduction networks or slow recovery if water molecules and reaction products are trapped inside the pores. Conversely, dense sputtered films are generally more reproducible and compatible with CMOS/MEMS processing, but they may provide insufficient internal surface area for strong gas interaction [79]. Thus, the optimal sensing layer is not necessarily the structure with the highest BET surface area; it is the architecture that simultaneously supports efficient gas diffusion, stable electron transport, sufficient receptor sites, and thermal/mechanical stability.

DFT and related calculations contribute to morphology design mainly by identifying surface facets, terminations, defect configurations, or pore-wall compositions that favor H_2_ activation, oxygen adsorption, and charge transfer. However, these molecular-scale descriptors alone cannot fully predict sensor performance, because the measured response also depends on film thickness, grain connectivity, pore accessibility, gas transport, and electrode geometry. Therefore, DFT insights should be combined with transport modeling and experimental characterization to connect surface reaction energetics with film-scale sensing behavior.

### 4.2. Noble-Metal Sensitization and Schottky Contacts

**Figure 16:**
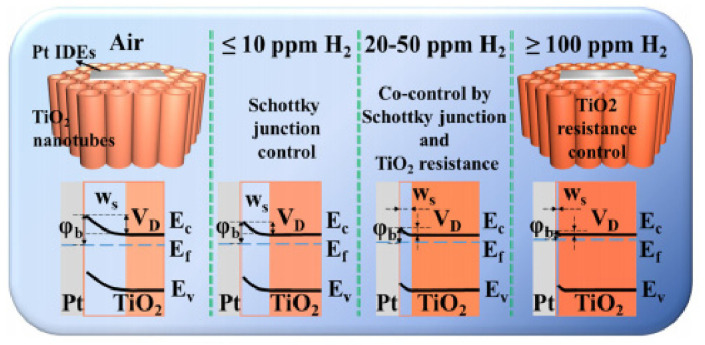
The primary mechanism driving the response of the TiO_2_ sensor in various atmospheres. All Panels adapted with permission from Ref. [75].

Noble metals such as Pd, Pt, Au, and Ag are widely used to enhance n-type MOS hydrogen sensors through chemical and electronic sensitization [12,23,24,48,49,50,51,52,53,54,55]. Chemical sensitization occurs when H_2_ molecules dissociate on noble-metal nanoparticles and the resulting atomic hydrogen spills over onto the oxide surface, where it reacts with chemisorbed oxygen species or directly modifies surface states. Electronic sensitization arises from work-function differences and Schottky-barrier formation at metal/oxide interfaces. The atmosphere-dependent response mechanism of the TiO_2_ Schottky-type hydrogen sensor is illustrated in Figure 16. In this case, hydrogen exposure can alter the work function, oxidation state, or hydride phase of the noble metal, thereby modulating the interfacial barrier height, oxide depletion layer, and overall sensor resistance [32,50,55,59,73,75].

Among noble metals, Pd is particularly important because it can selectively absorb hydrogen and form PdH_x_. In Pd-decorated ZnO, SnO_2_, or In_2_O_3_ sensors, the oxide generally serves as the high-surface-area transduction layer, while Pd provides efficient hydrogen dissociation and spillover [18,37,42,78]. This division of functions is especially effective when Pd nanoparticles are well dispersed and strongly coupled to the oxide surface. However, Pd-based systems also have limitations, including oxygen inhibition, hysteresis associated with Pd/PdH_x_ phase changes, volume expansion, catalyst sintering, and long-term baseline drift. Alloying Pd with other metals, reducing the catalyst size, improving nanoparticle dispersion, and using stable oxide supports can mitigate some of these issues. Nevertheless, such strategies should be evaluated under realistic conditions, including humid air, repeated cycling, and long-term operation, rather than only in dry single-gas measurements [10].

DFT and related first-principles calculations are useful for guiding noble-metal catalyst selection because they can compare H_2_ adsorption energies, H–H dissociation barriers, hydrogen diffusion barriers, work-function shifts, hydride formation tendencies, and interfacial charge transfer for Pd, Pt, Au, Ag, and bimetallic nanoparticles. These descriptors help clarify whether a catalyst primarily improves hydrogen activation, modifies the metal/oxide barrier, or changes the electronic structure of the oxide surface. For ZnO-based sensors, such calculations should also be connected to the hydrogen-induced metallization framework, because noble metals may not only dissociate H_2_ but also promote local hydrogenation and electronic reconstruction of ZnO surface states. To improve practical relevance, computational screening should include oxygen- and water-covered surfaces, catalyst oxidation states, and realistic metal/oxide interfaces rather than relying only on ideal dry surfaces.

A practical difficulty is to determine, for a given metal/oxide pair, whether the chemical (spillover) or the electronic (work-function/Schottky) pathway dominates, since the two often operate together. Several experimental and computational signatures help to deconvolve them. Electronic sensitization [9,10,11,23,24] is characterized by a response whose temperature dependence and apparent activation energy track the interfacial barrier height, by a correlation between work-function measurements and the sensor signal, and by Pd/PdH_x_ phase-related hysteresis detectable in X-ray diffraction and X-ray photoelectron spectroscopy. Chemical sensitization [12,18,19,32,37,50,55,73,75], in contrast, is governed by the catalyst/oxide perimeter and therefore scales with noble-metal particle size and dispersion rather than with bulk loading; it can be probed by exposing the device to pre-dissociated atomic hydrogen, which bypasses the metal dissociation step, and the interfacial and grain/grain-boundary contributions can be separated by impedance spectroscopy. Computationally, the two modes can be distinguished by comparing interfacial charge transfer (e.g., Bader analysis) and work-function shifts against H_2_ adsorption and dissociation energetics and spillover diffusion barriers. In practice, a convergent reading of these signatures—rather than any single measurement—is required to assign the dominant sensitization mode, and the diagnostic descriptors in Table 2 provide a consistent framework for doing so.

### 4.3. Defect and Dopant Engineering

Oxygen vacancies play a central role in n-type MOS hydrogen sensing because they influence oxygen adsorption, baseline conductivity, surface redox activity, and band bending [80,81]. In many oxide sensors, vacancies act as active sites for oxygen chemisorption and can modify the density of surface states that participate in charge transfer during hydrogen exposure. Therefore, controlling the type, concentration, and spatial distribution of defects is a key strategy for improving hydrogen response.

**Figure 17:**
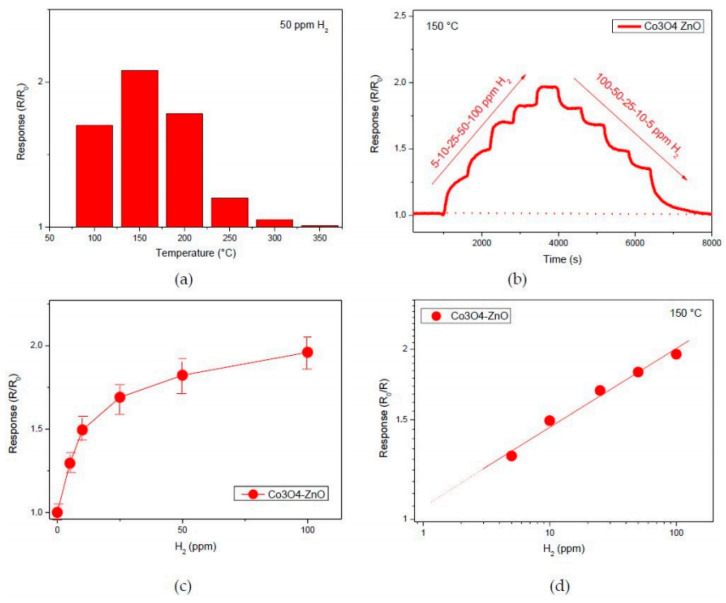
(**a**) Response vs. H_2_ concentration of the Co-doped ZnO sensor recorded at different temperatures. (**b**) Response to increasing and decreasing H_2_ concentration values at the temperature of 150 °C. (**c**) Calibration curve in a linear scale. (**d**) Calibration curve in a log-log scale. All Panels adapted with permission from Ref. [40].

Doping is one of the most widely used approaches for defect engineering. Appropriate dopants can increase the oxygen-vacancy concentration, reduce grain size, create additional adsorption sites, tune carrier density, and modify the electronic structure of the host oxide [82]. In ZnO, for example, Al, Co, Cd, and other cations can substitute for Zn^2+^ sites and introduce structural defects such as oxygen vacancies, zinc vacancies, or lattice distortion. Differences in ionic radius and valence state between Zn and the dopant can alter the lattice parameters, band structure, and surface defect chemistry of ZnO. Figure 17 summarizes the H_2_ response and calibration behavior of Co-doped ZnO sensors at different operating temperatures [40]. These changes can increase the number of adsorption-active sites and widen the depletion layer, thereby improving H_2_-sensing performance [36,83,84].

However, the beneficial effect of doping is usually limited to an optimum concentration range. Insufficient dopant content may not significantly change the surface chemistry or electronic structure, whereas excessive doping can increase baseline conductivity, reduce the relative resistance modulation, block active sites, form secondary phases, or destabilize the oxide lattice. Yang et al. reported Cd-doped ZnO nanorods for hydrogen sensing and showed that Cd doping can tune the crystal structure and nanorod morphology while improving the stability and H_2_-sensing performance of ZnO [36]. This example illustrates that dopants can affect both surface chemistry and microstructure, and these two effects should be considered together when interpreting sensor performance.

DFT provides useful descriptors for rational dopant selection, including dopant substitution energy, oxygen-vacancy formation energy, O_2_ and H_2_ adsorption energies, charge transfer between adsorbates and the oxide surface, band-edge shifts, and changes in the density of states. These calculations are particularly valuable for multicomponent oxides and doped composite systems, where multiple cation sites, oxidation states, and defect configurations can produce complex vacancy energetics [41]. For practical relevance, computational studies should compare not only ideal dry surfaces but also oxygen-covered, hydrogen-exposed, and water-containing surfaces, because humidity often strongly affects adsorption competition, baseline drift, and recovery behavior in real sensing environments.

### 4.4. N-N Heterojunctions and Multicomponent Composites

N–n heterojunctions are formed by combining two n-type oxides with different work functions, carrier concentrations, and band positions. When these oxides come into contact, charge transfer occurs until their Fermi levels are aligned, producing interfacial accumulation or depletion regions. Under hydrogen exposure, the sensor response is governed not only by modulation of the outer surface depletion layer but also by changes in the interfacial barrier. This additional barrier modulation can amplify the resistance change and improve the sensitivity compared with single-oxide sensors. This principle explains why SnO_2_–ZnO, In_2_O_3_–ZnO, SnO_2_–In_2_O_3_, and WO_3_–In_2_O_3_ structures often exhibit enhanced hydrogen-sensing performance [23,31,32,39,63,72,73].

**Figure 18:**
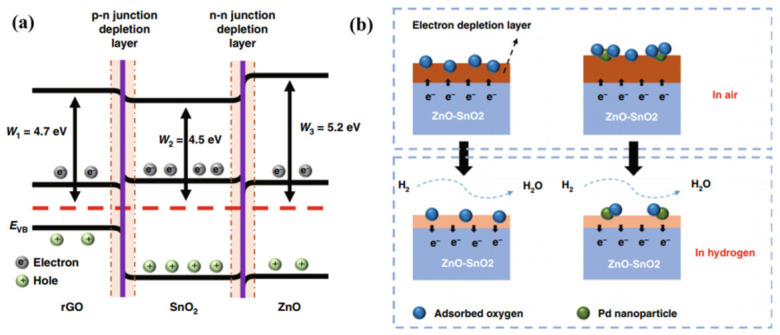
Gas sensing mechanism of Pd-doped rGO/ZnO-SnO_2_ based sensor. (**a**) Energy band structures of the rGO/ZnO-SnO_2_ p-n-n heterojunction (**b**) Gas sensing mechanism action diagram of Pd nanoparticles. All Panels adapted with permission from Ref. [38].

The effectiveness of n–n heterojunctions depends strongly on grain size, interface density, and the continuity of electron-transport pathways. Smaller grains can increase the number of homojunctions and heterojunctions, thereby providing more barrier-modulation sites. In SnO_2_–ZnO systems, for example, ZnO can contribute hydrogen-sensitive surface modulation and, under suitable conditions, metallization-related selectivity, whereas SnO_2_ provides strong oxygen-chemisorption-based transduction and stable charge transport [32]. Pd-functionalized In_2_O_3_–ZnO nanofibers introduce an additional catalytic function: Pd promotes hydrogen dissociation and spillover, In_2_O_3_/ZnO interfaces modulate interfacial barriers, and the porous nanofiber network facilitates gas accessibility [66]. Similarly, Pd-decorated In_2_O_3_ nanoparticle-embedded SnO_2_ porous nanofibers have been reported for enhanced hydrogen sensing. The energy-band structure and Pd-assisted gas-sensing mechanism of the rGO/ZnO–SnO_2_ heterojunction sensor are schematically illustrated in Figure 18. In this architecture, the enlarged surface area, increased grain-boundary density, synergistic interaction between SnO_2_ and In_2_O_3_ grains, and catalytic effect of Pd nanoparticles jointly improve the sensing performance [38].

DFT and electronic-structure calculations can support heterojunction design by predicting work functions, band offsets, interfacial charge transfer, adhesion energy, defect formation energies, and oxygen-vacancy localization. These descriptors are useful for identifying oxide pairs that can generate favorable interfacial barriers and stable charge-transfer pathways. However, heterojunction-based sensors usually cannot be explained by a single descriptor, because their performance results from the combined effects of catalytic activation, surface depletion-layer modulation, interfacial barrier control, percolated conduction, and gas diffusion. Therefore, computational analysis should be integrated with experimental tools such as impedance spectroscopy, Kelvin-probe measurements, temperature-programmed desorption, X-ray photoelectron spectroscopy, and operando spectroscopy to establish a more complete structure–interface–response relationship.

### 4.5. Low-Temperature Activation

Room-temperature or near-room-temperature hydrogen detection is a major goal for safety monitoring and low-power sensor operation. Several strategies have been explored to reduce the operating temperature of n-type MOS hydrogen sensors, including noble-metal catalysis [12,18,19,65], annealing treatment [45,85], UV illumination [86], heterojunction design [31,62], and highly porous nanostructures [38,82]. These approaches aim to compensate for the insufficient thermal activation of hydrogen dissociation, oxygen chemisorption, surface reaction kinetics, and charge transfer at low temperature.

However, lowering the operating temperature often introduces additional challenges. Slow surface reaction kinetics can lead to delayed recovery, while water adsorption becomes more competitive and can cause humidity-induced baseline drift. Therefore, low-temperature performance should not be evaluated only by response magnitude. Recovery time, response/recovery reversibility, baseline stability, selectivity, humidity tolerance, and long-term cycling stability should also be considered when assessing practical applicability.

Annealing treatment is one route to improve stability and response kinetics. Zhang et al. reported the effect of annealing treatment on the response characteristics of a Pd–Ni alloy-based hydrogen sensor [85]. Air annealing enhanced the stability of the baseline resistance and reduced zero drift. The authors found that both grain size and oxide content increased with annealing temperature, which improved the response rate and reduced variations in the response behavior. They also proposed that annealing can improve resistance to oxygen interference by modifying the microstructure and surface oxidation state of the Pd–Ni sensing layer.

**Figure 19:**
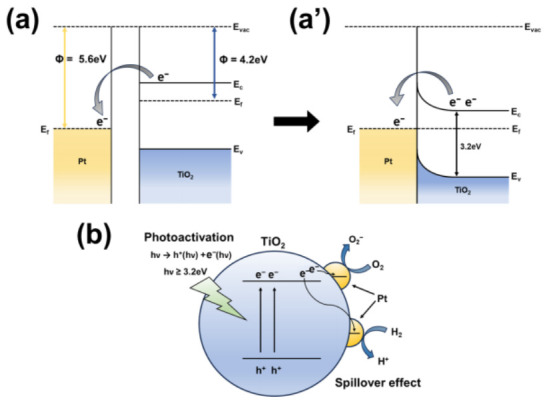
Schematics of the mechanism explaining the effects of UV illumination and Pt NPs on sensitivity enhancement. (**a**) Energy-band diagram before the contact between Pt and TiO_2_. (**a’**) Energy-band diagram after contact, where a Schottky barrier forms at the Pt/TiO_2_ interface, preventing electron recombination with holes on the TiO_2_ surface and facilitating charge separation. (**b**) Schematic of the hydrogen-sensing enhancement mechanism via the spillover effect of Pt NPs. All Panels adapted with permission from Ref. [86].

Photoactivation provides another pathway for low-temperature hydrogen sensing. UV-assisted hydrogen sensing has been demonstrated using Pt-nanoparticle-decorated TiO_2_ nanorods. Under 368 nm UV irradiation, the TiO_2_ nanorod/Pt nanoparticle sensor exhibited a response of 2.40 toward 1% H_2_, corresponding to an approximately 5.9-fold enhancement compared with pristine TiO_2_ nanorods measured in the dark [86]. The effects of UV illumination and Pt nanoparticle decoration on the H_2_-sensing mechanism of TiO_2_ nanorods are schematically shown in Figure 19. The enhanced performance was attributed to the synergistic effects of Pt catalytic activity and UV-induced photoactivation. Pt nanoparticles promoted hydrogen activation, increased interfacial charge modulation, and contributed to depletion-layer formation, while UV irradiation generated electron–hole pairs that facilitated surface reactions. This combined catalytic–photoactivated strategy helps overcome the limited activation energy available for hydrogen detection under ambient or low-temperature conditions.

Beyond the established routes above, several promising but still underexplored avenues may enable genuine room-temperature operation. Visible-light and plasmonic photoactivation could extend the UV-photoactivation concept to lower-energy, lower-power excitation [86]. Self-heating micro-hotplates and temperature-modulation schemes can deliver room-temperature-equivalent activation at low average power while simultaneously providing humidity-robust, information-rich responses [87]. Single-atom catalysts, by maximizing atomic efficiency and exposing uniform active sites, offer a route to efficient room-temperature spillover with minimal noble-metal loading [12,78,88]. Mixed-dimensional interfaces that couple metal oxides with two-dimensional materials such as MXenes or transition-metal dichalcogenides may provide highly tunable, low-temperature charge-transfer pathways [89,90]. Moisture-assisted surface protonic conduction, amplified by p–n heterojunction barriers, enables selective room-temperature H_2_ response without conventional high-temperature activation [31]. Finally, Field-effect (gate-tunable) transduction can amplify small surface-potential changes without thermal activation [71]. The principal open challenges for these approaches are the long-term stability of dispersed active sites, reproducible large-area fabrication, and reliable operation under realistic humid, multi-gas conditions.

### 4.6. Humidity Effects and Mitigation Strategies

Humidity is one of the most persistent obstacles to reliable n-type MOS hydrogen sensing, and its effects are mechanistically distinct from the target hydrogen reaction. In humid air, water molecules compete with oxygen and hydrogen for adsorption sites, promote surface hydroxylation, and modify the surface electron-depletion layer [44,69,72,73,80]. For many n-type oxides, water vapor can react with chemisorbed oxygen species and release electrons into the semiconductor, thereby decreasing the baseline resistance [44,69]. Consequently, humidity can either enhance or suppress the apparent hydrogen response depending on operating temperature, surface defect chemistry, catalyst state, and the relative changes in R_a_ and R_g_ [35,41,69,72,80]. At and near room temperature, chemisorbed water can also support protonic transport through hydrogen-bonded hydroxyl/water networks, including Grotthuss-type proton hopping, which introduces an additional humidity-dependent conduction pathway [44]. These processes alter both the baseline resistance and the surface reaction kinetics, typically resulting in baseline drift, reduced repeatability, and slower recovery [39,44,73,80]. The impact is particularly severe for low-temperature and high-surface-area sensors because water adsorption and desorption are less effectively suppressed and a larger fraction of the conduction pathway is exposed to adsorbed species [39,69,73,80].

Recent studies illustrate that humidity tolerance is material- and architecture-dependent. In Pd/Ce-In_2_O_3_ nanofibers, water vapor was reported to react with adsorbed O-species to form hydroxyl radicals and release electrons, while Ce^3+^/Ce^4+^ redox cycling, Pd-assisted catalytic reaction acceleration, and hydrophobic nanofiber surfaces contributed to enhanced anti-humidity behavior [69]. In Ag/Cu-In_2_O_3_ sensors, the response and baseline resistance decreased only slightly as the relative humidity increased from 32% to 85%, which was attributed partly to operation at 300 °C, where adsorbed water is more effectively removed from the sensing surface [35]. Similarly, Pd/WO_3_-SnO_2_ nanotube sensors were evaluated from 15% to 90% RH, and although the response gradually decreased because water molecules occupied oxygen vacancies and active sites through hydroxyl formation, a high response was still maintained at 90% RH [72]. For Pd-WO_3_/WS_2_ ternary nanocomposites, increasing humidity decreased both baseline resistance and response because hydroxyl poisoning of active sites donated electrons to the sensing layer and reduced the hydrogen response [73]. In contrast, Pd-SnO_2_-Co_3_O_4_ heterostructures retained only 38.8% of their initial response under 90% RH compared with 15% RH, directly demonstrating that water adsorption can block active sites for hydrogen reaction under harsh humid conditions [80]. DFT-assisted Sn-doped ZnO work further indicates that dopant selection can alter oxygen and hydrogen reaction energetics, electron release, and H_2_O adsorption strength, suggesting that humidity tolerance should be considered together with adsorption energetics and charge-transfer descriptors [41]. These examples show that humidity tolerance must be evaluated quantitatively rather than assumed from high dry-air response values [35,41,46,69,72,73,80].

Several complementary approaches can be used to mitigate humidity interference (Table 5). Hydrophobic coatings and molecular-sieving layers can suppress water and larger interfering gases while allowing H_2_ diffusion, although they may increase diffusion resistance and slow response/recovery [69,91,92]. Defect and dopant engineering can tune water-adsorption energetics, oxygen-vacancy density, and electron-transfer pathways; however, excessive defect density may also increase water sensitivity or baseline drift [41,69,80]. Noble-metal or bimetallic catalysts can accelerate hydrogen dissociation and surface redox reactions, helping the target reaction outcompete water-related processes [35,46,72,73,80]. Pore-size and morphology control can balance gas accessibility with water retention, whereas elevated-temperature, self-heated, or temperature-modulated operation can reduce water accumulation at the expense of power consumption and system complexity [35,41,44,72,73]. For practical deployment, humidity correction using calibration models, reference sensors, or sensor arrays should be combined with materials-level strategies, and sensor performance should be reported under realistic humid-air conditions using explicit RH values, baseline-stabilization protocols, and repeated exposure/recovery cycles [64,68,70,71,78]. Therefore, robust humid-air operation generally requires co-optimization of surface chemistry, selective diffusion barriers, catalyst stability, and device-level compensation rather than reliance on a single modification.

### 4.7. Representative Sensor Performances

Several trends and apparent anomalies in Table 6 warrant comment. First, the reported response and recovery times for nominally similar material classes vary by orders of magnitude; for example, Pd-decorated SnO_2_ and ZnO systems span from a few seconds to several minutes. Much of this variation does not reflect intrinsic material properties but rather differences in test-chamber volume, gas-flow rate, and baseline-stabilization protocol, which strongly influence the apparent kinetics. Second, the very slow recovery reported for some room-temperature devices (e.g., 48/862 s for the MOF-derived Pd@SnO_2_ composite) is consistent with sluggish low-temperature surface kinetics and competitive water adsorption as much as with intrinsic deep adsorption or PdH_x_ hysteresis. Third, the response magnitudes are not directly comparable because they are defined inconsistently (R_a_/R_g_, percentage change, or current ratio) and measured over widely different concentrations, temperatures, and humidities. The frequent absence of key parameters (entered as ‘null’) further limits quantitative comparison. These observations indicate that the apparent superiority of one system over another may partly reflect undefined testing conditions rather than genuine performance differences, and they motivate the minimum reporting standard proposed in Section 5.

### 4.8. DFT for Hydrogen Sensing: Current Limitations and Emerging Directions

Most reported DFT studies of hydrogen sensing model idealized [35,41,46,69,72], dry slabs at 0 K with a single adsorbate, and their conclusions can be sensitive to the choice of exchange–correlation functional, van der Waals correction, and Hubbard U. Such models cannot capture the competition of ubiquitous water vapor for adsorption sites, the dynamic formation of hydroxyl species, or the finite-temperature, entropic, and solvent effects that govern real humid-air operation. Emerging approaches are beginning to close this gap, including ab initio thermodynamics that map surface phase stability against oxygen and water chemical potentials, ab initio molecular dynamics and metadynamics for finite-temperature surface chemistry, grand-canonical (constant-potential) DFT, implicit-solvation models, machine-learning interatomic potentials that enable large-scale and long-time simulation of wet, defective surfaces, and the coupling of DFT with microkinetic modeling. Future computational screening for hydrogen sensors should therefore incorporate oxygen- and water-covered surfaces, realistic metal/oxide interfaces, and catalyst oxidation states rather than relying solely on ideal dry surfaces.

## 5. Challenges and Future Perspectives

First, high operating temperature remains a central limitation for n-type MOS hydrogen sensors. Many high-performance ZnO-, SnO_2_-, and In_2_O_3_-based sensors still operate in the range of 200–400 °C, which increases power consumption and can also activate responses to interfering gases. Noble-metal sensitization, UV activation, self-heated nanowires, heterojunction engineering, and high-surface-area porous oxides can reduce the required operating temperature. However, low-temperature operation should not be judged by response magnitude alone, because reduced thermal energy often slows recovery and increases susceptibility to humidity-induced drift. Therefore, practical low-temperature sensors should be evaluated using a combined performance set that includes response, recovery, baseline stability, selectivity, humidity tolerance, and cycling durability.

Second, selectivity remains difficult to achieve because many reducing gases interact with chemisorbed oxygen species and can produce resistance changes similar to those induced by hydrogen. This shared oxygen-consumption pathway limits the intrinsic selectivity of many single-component MOS sensors. Future sensor designs should therefore combine multiple selectivity mechanisms, including chemical selectivity from Pd or bimetallic catalysts, physical selectivity from molecular-sieving layers, and electronic selectivity from heterojunction or Schottky-barrier modulation. The most robust designs will likely rely on the integration of several complementary mechanisms rather than on a single material modification.

Although selectivity is frequently addressed by invoking sensor arrays, the material strategies reviewed here can be used deliberately to construct such arrays rather than merely to pursue a single perfect sensor. By systematically varying the catalyst (Pd, Pt, Au, or bimetallic), the dopant, the n–n heterojunction partner, the operating temperature, and the presence of molecular-sieving layers, one can assemble a set of elements with deliberately orthogonal cross-sensitivities. The resulting multidimensional response vectors can then be resolved into hydrogen identification and interferent compensation using multivariate pattern recognition such as principal-component analysis or machine learning. Temperature modulation of a single element can generate an equivalent virtual array, reducing footprint and power. Effective array design should therefore be co-developed with low-power readout and data-processing electronics, so that the material diversity reviewed here is translated into discriminating, application-specific response patterns.

Third, standardized reporting is essential for meaningful comparison across studies. Key experimental parameters, including response definition, chamber volume, gas-flow rate, hydrogen concentration, background gas, relative humidity, operating temperature, baseline stabilization time, response/recovery-time definition, LOD calculation method, and long-term drift protocol, should be explicitly reported. Without such standardization, the apparent superiority of one material or architecture over another may reflect differences in testing protocol rather than intrinsic sensing performance.

To make the call for standardization actionable, we propose a minimum reporting checklist; meaningful cross-study comparison requires the following parameters to be reported explicitly: (i) material composition, synthesis method, morphology, film thickness, and substrate/electrode configuration; (ii) the explicit definition of the response (Ra/Rg, ΔR/Ra, ΔG/Ga, etc.); (iii) the H_2_ concentration range and balance gas (air or synthetic air with stated O_2_ %, or N_2_); (iv) the relative humidity and the reference temperature at which it was determined; (v) the operating temperature; (vi) the definition of response and recovery time (e.g., 90%/10% criterion); (vii) the test-chamber volume and gas-flow rate; (viii) the baseline-stabilization protocol and duration; (ix) the limit-of-detection calculation method (signal-to-noise basis); (x) repeatability (number of cycles) and the long-term drift protocol and duration; (xi) the selectivity test gases, concentrations, and whether they were tested under humid conditions; and (xii) the measurement circuit and noise statistics where available.

The translation of high-performance nanomaterials into miniaturized, low-power devices is constrained by their compatibility with standard microfabrication. Sputtered and atomic-layer-deposited films are directly compatible with CMOS/MEMS micro-hotplate processing and offer good reproducibility and thermal stability, but they may provide limited internal surface area and hence lower intrinsic sensitivity. Conversely, electrospun, hydrothermal, and MOF-derived nanostructures deliver high sensitivity and rich surface chemistry but are difficult to deposit locally, to pattern, and to integrate within the thermal budget and contamination constraints of back-end-of-line processing. Bridging this integration gap is an active area of research, with promising routes including localized hydrothermal growth on pre-patterned electrodes, inkjet and aerosol-jet printing of functional inks, direct growth on suspended micro-hotplates, and low-thermal-budget post-CMOS integration. Closing this gap is essential if laboratory-scale sensitivity is to be retained in manufacturable, low-power hydrogen-safety devices.

### Emerging Materials and Transduction Beyond Conventional N-Type MOS

Recent review articles published in 2024–2026 indicate that hydrogen and gas-sensing research is rapidly expanding beyond the conventional n-type MOS paradigm based solely on oxygen chemisorption, electron-depletion-layer modulation, and surface redox reactions [4,79,93,94,95,96,97,98,99]. Although n-type MOS materials such as ZnO, SnO_2_, In_2_O_3_, WO_3_, and TiO_2_ remain central platforms for chemiresistive hydrogen sensing, recent studies increasingly emphasize new material systems, porous architectures, and hybrid transduction concepts that enable lower operating temperature, improved selectivity, and better environmental robustness [4,95,97,99].

Two-dimensional nanomaterials, including graphene, transition metal dichalcogenides, and MXenes, have emerged as attractive sensing components because of their atomically thin conduction channels, large surface-to-volume ratios, tunable band structures, and rich surface chemistry [93,96]. Unlike conventional MOS sensors that generally require thermally activated oxygen adsorption and desorption, 2D materials can respond through direct charge transfer, adsorption-induced Fermi-level shifts, interfacial Schottky barrier modulation, and surface-functional-group-mediated gas adsorption [93]. MXenes are particularly promising because their high electrical conductivity, hydrophilic surface terminations, solution processability, and flexible-device compatibility allow their integration into low-power and wearable sensing platforms [96].

Single-atom catalyst-modified metal oxides represent another important emerging direction. Isolated Pt, Pd, Au, Rh, Ni, Ag, or Cu atoms anchored on oxide surfaces maximize atomic utilization and provide uniform catalytic sites for gas activation [94]. For hydrogen sensing, such single-atom sites may promote H_2_ dissociation, spillover, oxygen activation, and interfacial charge transfer with minimal noble-metal loading. Compared with conventional noble-metal nanoparticles, single-atom modifiers offer more precise active-site structures, although their practical use still requires improved high-loading synthesis, aggregation resistance, long-term stability, and scalable fabrication [94].

Recent hydrogen-sensor reviews also highlight Pd-based nanostructures, Pd alloys, and Pd-based composites as material systems that operate through mechanisms distinct from MOS surface depletion [95]. In these sensors, hydrogen detection can arise from reversible PdH_x_ formation, resistivity modulation, nanogap closure, volume expansion, or phase-transition control, enabling room-temperature or near-room-temperature operation [98]. In parallel, MOS–2D material hybrids, MOS–conducting polymer composites, and multicomponent heterostructures are being explored to combine the strong surface reactivity of oxides with the high carrier mobility, flexibility, and low-temperature response of emerging conductive materials [4,97].

Porous materials, including metal–organic frameworks, covalent organic frameworks, porous organic polymers, and their oxide hybrids, provide another important route for extending the conventional MOS concept [79]. Their high specific surface area, tunable pore size, ordered channels, and functional groups can improve gas adsorption, molecular sieving, and selectivity. In hydrogen sensing, MOF/MOS and MOF/Pd hybrid structures can act as selective diffusion or nanofiltration layers, allowing small H_2_ molecules to access active sites while suppressing larger interfering gases. Therefore, porous materials are increasingly used not only as sensing materials but also as selectivity-enhancing overlayers and interface modifiers [79].

From a practical deployment perspective, recent 2026 reviews also emphasize that next-generation hydrogen sensors should not be evaluated only by material sensitivity under ideal laboratory conditions. Realistic operation requires resistance to humidity, temperature fluctuation, cross-sensitive gases, poisoning, and long-term drift [98]. In this context, machine-learning-assisted semiconductor metal oxide hydrogen sensing has emerged as a complementary strategy for sensor calibration, drift compensation, noise suppression, concentration estimation, selectivity enhancement, sensor fusion, real-time monitoring, predictive maintenance, and material optimization [99]. These algorithmic approaches are particularly useful because chemiresistive sensors often show nonlinear and environment-dependent responses under humid or mixed-gas conditions.

Overall, the field is shifting from single-material n-type MOS chemiresistors toward integrated material–interface–device–algorithm systems. In future hydrogen sensors, oxide nanostructures, single-atom catalysts, 2D conductive channels, porous molecular-sieving layers, selective coatings, sensor arrays, and machine-learning-based compensation are expected to be co-designed. This transition will be essential for achieving room-temperature operation, low power consumption, high selectivity, humidity tolerance, and long-term reliability in practical hydrogen-energy infrastructure.

## 6. Conclusions

N-type metal oxide semiconductor hydrogen sensors provide a versatile and manufacturable platform for hydrogen safety monitoring. ZnO, SnO_2_, and In_2_O_3_ remain central materials in this field because they combine surface oxygen chemistry, tunable electronic transport, rich nanostructuring capability, and compatibility with catalytic and heterojunction engineering. Each material offers distinct advantages: ZnO is notable for hydrogen-sensitive surface modulation, including possible H-induced metallization under suitable conditions; SnO_2_ provides a mature commercial base and strong oxygen-chemisorption-based transduction; and In_2_O_3_ offers high electron mobility and defect-sensitive surface chemistry.

The most effective sensor architectures increasingly rely on the integration of multiple enhancement mechanisms rather than on a single material modification. High-surface-area morphologies improve gas accessibility, noble-metal catalysts promote H_2_ dissociation and spillover, dopant-induced oxygen vacancies tune adsorption and carrier transport, n–n heterojunctions amplify interfacial barrier modulation, and molecular-sieving or hydrophobic layers improve selectivity and environmental stability. DFT and related first-principles calculations are most valuable when used as distributed design tools within these strategies. Descriptors such as adsorption energy, vacancy formation energy, hydrogen dissociation barriers, work-function shifts, band alignment, and interfacial charge transfer can guide catalyst selection, defect engineering, interface design, and selective barrier-layer development.

Despite substantial progress, no n-type MOS hydrogen sensor simultaneously satisfies all practical requirements, including low-temperature operation, sub-ppm sensitivity, fast response and recovery, high selectivity, humidity tolerance, low drift, and long-term stability. Future progress will therefore require the co-design of sensing materials, catalytic interfaces, microstructure, device architecture, and validation protocols. CMOS/MEMS integration will be important for miniaturization, low-power operation, and scalable manufacturing, but it should be regarded as an enabling platform rather than a substitute for robust materials and interface engineering. Ultimately, the transition from high-performance laboratory sensors to reliable hydrogen safety devices will depend on standardized testing, realistic humid-air operation, long-term cycling data, and mechanistic understanding across both surface chemistry and device-level transport.

To make the call for co-design concrete, we highlight three specific, emerging research themes: (1) catalyst–support–microstructure co-design, in which DFT, microkinetic, and charge-transport modeling are combined with fabrication-compatible micro-hotplate platforms to guide computation-informed yet manufacturable sensing layers; (2) selectivity-by-design arrays, in which libraries of deliberately cross-sensitive elements (varied catalysts, molecular sieves, and heterojunctions) are co-developed with machine-learning pattern recognition and low-power readout electronics; and (3) operando-validated materials design, in which operando spectroscopy (e.g., DRIFTS, ambient-pressure XPS, and impedance spectroscopy) is coupled with realistic, humid, multi-gas DFT/AIMD in a closed loop to build predictive structure–interface–response models.

## Figures and Tables

**Table 1:** Common performance metrics for n-type MOS H_2_ sensors.

Metric	Common Definition	Why It Matters	Caution for Comparison
Response	R_a_/R_g_ for n-type reducing gases; sometimes ΔR/R_a_ or ΔG/G_a_	Primary indicator of transduction strength	Values depend strongly on concentration, humidity, operating temperature, and baseline resistance.
Response time	Time to reach 90% of steady-state signal	Safety-critical for leak detection	Often affected by chamber volume and gas-flow protocol.
Recovery time	Time to return to 10% of peak signal after H_2_ removal	Determines reusability and duty cycling	Slow recovery can arise from deep adsorption, hydride formation, or water retention.
LOD	Concentration estimated at signal/noise threshold	Early leakage detection	Must include noise statistics and calibration range.
Selectivity	Relative response to H_2_ versus interfering gases	Essential for practical deployment	Should be tested in realistic mixtures and humidity, not only in dry single-gas tests.
Stability	Signal retention over cycles, days, or months	Commercial readiness	Accelerated aging, humidity cycling, and catalyst sintering must be considered.

**Table 2:** Relative contributions of the principal n-type MOS hydrogen-sensing mechanisms and the conditions under which each becomes dominant.

Mechanism	Dominant Operating Regime	Primary Contribution to the Signal	Diagnostic Signature (Experimental/Computational)	Representative Systems [Ref.]
Oxygen chemisorption (depletion-layer modulation)	All n-type oxides; the baseline mechanism. Most effective at intermediate temperature (~150–400 °C); suppressed at very low T (weak activation) and very high T (desorption), giving the volcano-shaped response–T curve.	Ionosorbed O_2_^−^/O^−^/O^2−^ deplete surface electrons in air; H_2_removes chemisorbed oxygen and returns electrons, narrowing the depletion layer and grain-boundary barriers and decreasing resistance. Universal transduction baseline.	Volcano-shaped response vs temperature; strong O_2_-partial-pressure dependence; broad cross-sensitivity to reducing gases; activation energy set by O-species chemistry.	Pristine SnO_2_, ZnO, In_2_O_3_ [9,10,11,20,27]
Catalytic spillover (chemical sensitization)	Noble-metal-decorated oxides (Pd, Pt, Au). Enables low-temperature/room-temperature operation. Scales with catalyst dispersion and metal/oxide perimeter; favors H_2_.	H_2_ dissociates on the metal; atomic H spills onto the oxide and accelerates reaction with chemisorbed oxygen, lowering the effective activation temperature and increasing response magnitude and speed.	Response and kinetics depend on catalyst loading, dispersion, and particle size (perimeter scaling); response retained at reduced T; sensitivity to pre-dissociated atomic H; lowered apparent activation energy.	Pd@SnO_2_ [12], Pd/SnO_2_ NWs [18], Pd–ZnO [19,37]
Heterojunction barrier modulation (n–n)	Composite/bilayer oxides (SnO_2_–ZnO, In_2_O_3_–ZnO, SnO_2_–In_2_O_3_, WO_3_–In_2_O_3_). Moderate-to-high T. Strengthens with interface density (smaller grains, tuned composition).	Fermi-level alignment forms interfacial barriers; their modulation under H_2_ amplifies the resistance change beyond single-oxide layers, coupled with surface depletion-layer modulation.	Response enhancement vs single-oxide control; impedance spectroscopy separating interfacial from grain/grain-boundary terms; band offsets from Kelvin-probe/work-function data; dependence on composition ratio.	SnO_2_–In_2_O_3_ [23], SnO_2_/ZnO [24,32,38], In_2_O_3_–ZnO [31], WO_3_–In_2_O_3_ [39]
Hydrogen-induced metallization (ZnO)	Highly condition-dependent (not universal): ultrafine ZnO grains, nonpolar surface terminations, Pd/Pt-assisted dissociation, elevated T, and a sufficiently reducing local environment.	Local reduction/hydrogenation forms quasi-metallic conductive surface states, an additional transduction pathway beyond depletion-layer modulation, and can raise H_2_ selectivity.	Unusually high H_2_ selectivity vs other reducing gases (notably vs SnO_2_ control); reversible reduction–reoxidation; ideally DOS/charge-delocalization changes near E_F (operando, currently limited). Hard to separate from vacancy donation, hydroxyl chemistry, or PdH_x_ (see Section 2.2).	NiO/ZnO NF [25], Pd@ZnO/SnO_2_ [32], Electrospun ZnO NFs [33],
Defect engineering (oxygen vacancies/doping)	Tunable across T; benefit confined to an optimum dopant/vacancy window. A cross-cutting modifier affecting baseline conductivity and active-site density.	Oxygen vacancies act as adsorption-active sites and modify surface states/band bending; aliovalent doping tunes carrier density, grain size, and vacancy concentration, widening the depletion layer and adding active sites.	Volcano-type dependence on dopant concentration; XPS O1s lattice/defect ratio correlation; EPR vacancy signals; baseline-resistance shifts; DFT vacancy-formation and adsorption energetics.	Zn–In_2_O_3_ [34], Ag/Cu–In_2_O_3_ [35], Cd:ZnO [36], Co:ZnO [40], Sn:ZnO [41]

T, temperature; NW, nanowire; NF, nanofiber; E_F, Fermi level; DOS, density of states; XPS, X-ray photoelectron spectroscopy; EPR, electron paramagnetic resonance; DFT, density functional theory.

**Table 5:** Comparison of anti-humidity strategies for n-type MOS hydrogen sensors.

Humidity-Related Issue	Mechanistic Origin	Mitigation Strategy	Trade-off or Caution	Refs.
Baseline drift/response variation	H_2_O competes with O_2_/H_2_ hydroxylation and depletion width.	Report R_a_/R_g_ versus RH; apply RH-dependent calibration.	Dry-air data may overestimate performance.	[35,44,69,72,73,80]
Water-induced electron donation	H_2_O can react with adsorbed oxygen, form–OH species, and release electrons.	Use catalysts/dopants that favor H_2_ reactions over water side reactions.	May mask the true H_2_ response.	[44,69]
Surface hydroxyl accumulation	Hydroxyl species occupy active sites and slow recovery.	Use redox-buffering additives such as Ce species.	Excess doping may reduce response.	[41,69,80]
Low-temperature protonic conduction	Adsorbed water can create a proton-conduction pathway.	Verify low-temperature signals under controlled RH.	May cause unstable apparent response.	[44,72,73,80]
Active-site blocking at high RH	H_2_O preferentially occupies reactive surface sites.	Apply hydrophobic/sieving layers or catalyst-rich interfaces.	Barrier layers can slow diffusion.	[35,41,69,91]
Elevated-temperature operation	Heating lowers water residence time on the surface.	Use optimized temperature, self-heating, or temperature modulation.	Higher power and thermal aging.	[35,46,72,73,80]
Catalyst/interface control	H_2_ spillover and activated oxygen improve H_2_ reaction kinetics.	Use Pd/PdOx, bimetallic catalysts, or heterointerfaces.	Check catalyst stability in humid cycling.	[44,72,73]
Defect/dopant tuning	Vacancies and dopants tune O_2_, H_2_, and H_2_O adsorption.	Optimize defect density; include water-covered DFT models.	Excess defects may increase drift.	[35,41]

**Table 6:** Representative n-type MOS hydrogen sensors.

System	N-Type Base	Strategy	H_2_ Condition	Temp.	Response	Response/Recovery	Ref.
50% O_2_-CVD-decorated SnO_2_	SnO_2_	CBD-grown SnO_2_ modified by oxygen-content-regulated CVD	100 ppm	350 °C	271%	2/29 s	[13]
0.05 wt% NiO-loaded ZnO nanofibers	ZnO	p-NiO/n-ZnO heterojunction, ZnO metallization, NiO catalytic effect	0.1–10 ppm	200 °C	null	null	[25]
Pd nanoparticle-decorated SnO_2_ nanowires	SnO_2_	Pd decoration by UV irradiation, Schottky junction, PdH_x_ formation	100 ppm	300 °C	56 (R_a_/R_g_)	22/164 s	[18]
Pd-functionalized ZnO nanowires	ZnO	Pd sensitization, PdH_x_ formation, ZnO metallization	100 ppm	350 °C	87.17 (R_a_/R_g_)	null	[19]
Annealed SnO_2_ thin film	SnO_2_	RF-sputtered SnO_2_ thin film, annealing-time optimization	5 vol%	300 °C	257.34%	3/null s	[45]
1.0% Pd/SnO_2_ ultrathin nanosheets	SnO_2_	Ultrathin nanosheets, Pd loading, DFT-supported catalytic sensitization	20 ppm	220 °C	75 (R_a_/R_g_)	21/13 s	[46]
Pd-decorated SnO_2_ nanofilm on Si nanowires	SnO_2_	SiNW substrate, Pd spillover, oxygen vacancies, Pd/SnO_2_ Schottky barrier	1.5 vol%	300 °C	>9	9/null s	[47]
4.0 at.% Au-loaded SnO_2_ nanoparticles	SnO_2_	Au loading, hydrothermal nanoparticles, chemical/electronic sensitization	100 ppm	250 °C	25 (R_a_/R_g_)	1/3 s	[48]
Pd_3_Pt nano-octahedron-modified SnO_2_	SnO_2_	Shape/composition-controlled PdPt bimetallic catalyst, room-temperature activation	1000 ppm	25 °C	22,821	1/8 s	[49]
13 wt% Pd@SnO_2_ porous composite	SnO_2_	MOF-derived porous SnO_2_, Pd decoration, oxygen vacancies	50 ppm	25 °C	25.4 (R_a_/R_g_)	48/862 s	[12]
Pd/ZnO–SnO_2_ hollow nanofibers	ZnO/SnO_2_	Pd decoration, hollow nanofibers, ternary Pd/ZnO/SnO_2_ heterojunctions	200 ppm	270 °C	171 (R_a_/R_g_)	19/<1 s	[50]
SnO_2_–In_2_O_3_ nanocomposite	SnO_2_/In_2_O_3_	Impregnation-derived SnO_2_ nanoclusters on In_2_O_3_, percolated SnO_2_ paths, In-induced oxygen vacancies	1100 ppm	300 °C	1400	≤1/null s	[23]
SnO_2_/ZnO heterojunction thin film	SnO_2_/ZnO	DC-sputtered n–n heterojunction, ZnO thickness optimization	100 ppm	270 °C	58.8%	3.7/127.3 s	[24]
Ag/ZnO hollow microstructures	ZnO	Hierarchical hollow ZnO, Ag chemical/electronic sensitization	300 ppm	250 °C	479%	175/655 s	[54]
Pd@ZnO/SnO_2_ bilayer thin film	ZnO/SnO_2_	Pd spillover, SnO_2_/ZnO heterojunction modulation, H_2_-induced ZnO metallization, CMOS-compatible sputtering	20 ppm–4 vol%	300 °C	2.49 (R_a_/R_g_)	<1/<3 s	[32]
Pd nanocube-decorated ZnO nanorod array	ZnO	Pd nanocube decoration, 1D ZnO nanorod array, Pd spillover/hydride-related effect	10,000 ppm	100 °C	≈74%	1.98/2.04 min	[57]
Pd@ZnO core–shell nanoparticles	ZnO	Pd@ZnO core–shell structure, metallic Pd core preservation, high BET surface area	100 ppm	350 °C	22 (R_a_/R_g_)	1.4/7.8 min	[58]
2% Ag-incorporated ZnO nanoparticles	ZnO	Ag incorporation, porosity modulation, spillover effect, surface metallization	H_2_ gas	—	≈4357%	4.3/6.5 s	[37]
1:1 Ag:Pd-decorated ZnO nanorods	ZnO	Bimetallic Ag–Pd nanoparticle decoration, hydrothermal ZnO nanorods, synergistic catalytic sensitization	100 ppm	275 °C	51.36 (R_a_/R_g_)	null	[60]
ZnO–SnO_2_ composite sensor	ZnO/SnO_2_	n–n heterojunction, chemically synthesized composite film, annealing optimization	10,000 ppm	150 °C	≈90%	60/75 s	[62]
NiO/ZnO nano-bulk heterostructure	ZnO/NiO	p–n heterojunction, surface protonic conduction through chemisorbed moisture	1200 ppm	RT	≈71 ± 20%	72/null s	[63]
Pd-functionalized In_2_O_3_-loaded ZnO nanofibers	ZnO/In_2_O_3_	Electrospun nanofibers, In_2_O_3_ loading, Pd functionalization, multiple heterojunctions, PdH_x_ formation	50 ppb	350 °C	172 (R_a_/R_g_)	null	[31]
Fern-like In_2_O_3_@ZnO@Pd nanotubes	In_2_O_3_/ZnO	MOF-template-derived nanotubes, Pd loading, fern-like morphology, room-temperature operation	10,000 ppm	RT	270 (R_a_/R_g_)	32/116 s	[66]
Au_1_/In_2_O_3_ single-atom catalyst	In_2_O_3_	Single-atom Au loading, mesoporous nanorod-like In_2_O_3_, electronic sensitization, oxygen vacancies	10 ppm	200 °C	31 (R_a_/R_g_)	null	[68]
Pd/Ce co-doped In_2_O_3_ nanofibers	In_2_O_3_	Rare-earth/noble-metal co-doping, orbital hybridization, oxygen-vacancy engineering, electrospun nanofibers	10 ppm	280 °C	41.13 (R_a_/R_g_)	<1/5 s	[69]
2 at% CeO_2_-loaded In_2_O_3_ hollow spheres	In_2_O_3_	CeO_2_ loading, hollow-sphere morphology, n–n heterojunction, oxygen-vacancy modulation	50 ppm	160 °C	20.7 (R_a_/R_g_)	1/9 s	[71]
2% Zn-doped In_2_O_3_ dendritic protrusion nanospheres	In_2_O_3_	Zn doping, hierarchical porous nanospheres, oxygen-vacancy enhancement	500 ppm	340 °C	24.6 (R_a_/R_g_)	2/3 s	[34]
WO_3_–C/In_2_O_3_ MEMS sensor	In_2_O_3_/WO_3_	MOF-derived porous In_2_O_3_, carbon coupling, WO_3_ decoration, multicomponent heterojunctions, MEMS integration	1000 ppm	250 °C	10.11 (R_a_/R_g_)	1.9/9.2 s	[39]
Pd/WO_3_–SnO_2_ nanotubes	SnO_2_/WO_3_	Pd decoration, WO_3_–SnO_2_ heterojunction, hydrogen spillover, oxygen-vacancy activation	50 ppm	90 °C	235.52 (R_a_/R_g_)	1/null s	[72]
2 at% Pd–WO_3_/WS_2_ ternary nanocomposite	WO_3_/WS_2_	Pd decoration, WO_3_/WS_2_ heterostructure, p–n–p heterojunction, electronic sensitization	1000 ppm	125 °C	4227.35 (R_a_/R_g_)	1/25 s	[73]
TiO_2_ rutile–anatase homojunction, TiO_2_-R/A-25 mL	TiO_2_	Rutile/anatase homojunction, porous TiO_2_ architecture, charge-transfer enhancement	2500 ppm	RT	1661 (R_a_/R_g_)	21/null s	[76]
Pd–SnO_2_–Co_3_O_4_ heterostructure, Pd-Sn-Co	SnO_2_/Co_3_O_4_	Pd decoration, SnO_2_–Co_3_O_4_ p–n heterojunction, oxygen-vacancy engineering, Co^3+^/Co^2+^ redox modulation	30 ppm	90 °C	100.6 (R_a_/R_g_)	6/40 s	[80]
8 mol% Co-doped ZnO nanorods	ZnO	Co doping, oxygen-vacancy generation, hydrothermal ZnO nanorods	3000 ppm	300 °C	≈99.2% [(I_g_ − I_a_)/I_a_] × 100	74/40 s	[40]

R_a_/R_g_ is the response, defined as the ratio of resistance in air (R_a_) to resistance in H_2_ (R_g_). The “Response” and “Response/recovery” columns list the reported response value and the response/recovery times, respectively; “null” indicates a value that was not reported in the cited study. Where only a response time was reported, the recovery field is given as “null” (e.g., 3/null s). LOD is the limit of detection. RT denotes room temperature, and “—” indicates a value that was not reported. The Pd–Ni alloy entries are included because they were discussed in the low-temperature activation/annealing context, although they are not n-type MOS materials; the porous-material review article was excluded because it is not an individual H_2_ sensor performance study.

## Data Availability

No new data were created or analyzed in this study. Data sharing is not applicable to this article.
